# Apoptotic cells for treatment of acute respiratory distress syndrome associated with COVID-19

**DOI:** 10.3389/fimmu.2023.1242551

**Published:** 2023-08-02

**Authors:** Peter Vernon van Heerden, Avraham Abutbul, Ahmad Naama, Shlomo Maayan, Nassar Makram, Akiva Nachshon, Kamal abu Jabal, Oren Hershkovitz, Lior Binder, Yehudit Shabat, Barak Reicher, Dror Mevorach

**Affiliations:** ^1^ General Intensive Care Unit, Hadassah-Hebrew University Medical Center, Jerusalem, Israel; ^2^ Medical Intensive Care Unit, Hadassah-Hebrew University Medical Center, Jerusalem, Israel; ^3^ Department of Emergency Medicine, Hadassah-Hebrew University Medical Center and Hebrew University-Hadassah Faculty of Medicine, Jerusalem, Israel; ^4^ Infectious Diseases Division, Barzilai Medical Center, Ashkelon, Israel; ^5^ Ziv Medical Center and Azrieli Faculty of Medicine, Bar Ilan University, Safed, Israel; ^6^ Enlivex Therapeutics Ltd., Ness Ziona, Israel; ^7^ Department of Medicine, Hadassah-Hebrew University Medical Center, Jerusalem, Israel; ^8^ The Institute of Rheumatology-Immunology-Rheumatology, The Wohl Institute for Translational Medicine, Hadassah-Hebrew University Medical Center and Hebrew University-Hadassah Faculty of Medicine, Jerusalem, Israel

**Keywords:** apoptotic cells, cytokine storm, ARDS, macrophage reprogramming, COVID-19

## Abstract

**Background:**

Hyper-inflammatory immune response, a hallmark of severe COVID-19, is associated with increased mortality. Acute respiratory distress syndrome (ARDS) is a common manifestation. We undertook two phase I/II studies in five and then 16 subjects with severe/critical COVID-19 to assess the safety and preliminary efficacy of apoptotic cells (Allocetra™-OTS, Enlivex Therapeutics), a cellular immunomodulatory therapy that reprograms macrophages to reduce hyper-inflammatory response severity.

**Methods:**

Eligible patients presenting to the Emergency Room with severe COVID-19 and respiratory dysfunction received one intravenous administration of Allocetra™-OTS and were monitored for adverse events (AEs) for 28 days. The primary aim was to determine the safety profile of treatment; secondary aims were recovery from ARDS, intensive care unit (ICU) and hospital length-of-stay, and mortality. Immune modulator markers were measured to elucidate the mechanism of action of Allocetra™-OTS.

**Results:**

21 patients with severe-critical COVID-19 of Gamma, Alpha and Delta variants, were treated with a single dose of apoptotic cells. 19/21 patients had mild-to-severe ARDS at presentation. Median age was 53 years, 16/21 were males, 16/21 were overweight/obese. No serious related adverse events (SAEs) were reported. All 21 study subjects survived to day 28 (end of study); 19/21 recovered completely. Comparable mortality rates at the hospital were 3.8%−8.9% for age- and gender-matched patients, and 39%−55% for critical patients. Recovering patients exhibited rapid ARDS resolution and parallel resolution of inflammation markers and elevated cytokines/chemokines.

**Conclusion:**

In patients with severe/critical COVID-19 associated with ARDS, Allocetra™-OTS was safe, well-tolerated, and showed promising results for resolution of respiratory failure and inflammation.

**Trial registration:**

https://clinicaltrials.gov/ct2/show/study/NCT04513470, https://clinicaltrials.gov/ct2/show/study/NCT04590053, Identifiers NCT04513470, NCT04590053.

## 1 Introduction

Severe acute respiratory syndrome coronavirus disease 2019 caused a global pandemic of coronavirus disease 2019 (COVID-19). Worldwide, the case fatality rate (CFR) of COVID–19 ranged between ~4% during the wild-type/alpha/delta virus infection period before the availability of vaccines, and ~1% during the dominance period of the Omicron SARS-COV-2 variants (1). During the wild-type/alpha/delta virus infection period, COVID-19 also resulted in complications among hospitalized patients, resulting in up to 15–25% of in-patients being admitted to the intensive care unit (ICU) (2–4). Acute respiratory distress syndrome (ARDS) is a severe pulmonary condition and one of the major complications of COVID-19 (5, 6). The pathogenesis of ARDS involves diverse immune cells of both the innate and adaptive immune systems that mediate the propagation of lung injury triggered by an insult such as severe viral infection. The resultant lung infiltrates and hypoxemia may be life-threatening. The immune response in COVID-19, which may result in ARDS, involves the unbalanced secretion of various inflammatory cytokines and chemokines, including TNF-α, IL-1β, IFN-γ, IL-6, IL-10, IL–1RA, IP–10, MCP–1, and IL-8 (CXCL8). These cytokines and chemokines were found to be independent and significant predictors of disease severity and death (7–10). Yang et al. showed that 81% of patients lost to COVID-19 also had ARDS (11). Moreover, a meta-analysis of 45 studies across 17 countries showed that more than 75% of COVID–19 patients admitted to the ICU developed ARDS (12).

This vigorous host response to infection, sometimes termed “cytokine storm,” is due to an immune system gone awry and an inflammatory response flaring out of control. This term has captured the attention of the public and the scientific community alike and is increasingly being used in both popular media and scientific literature. Indeed, recent publications have indicated that many of the complications of COVID-19 are related to the cytokine storm (13, 14).

In a previous clinical study using apoptotic cells (Allocetra-OTS, Enlivex Therapeutics, Israel) for immune modulation (ClinicalTrials.gov Identifier: NCT03925857), we observed that administration of Allocetra-OTS to patients with sepsis was safe and had a significant immuno-modulating effect, leading to resolution of the cytokine storm. All patients were alive at the end of the study. Based on comparisons with mortality score prediction and historical matched-controls, and the resolution of organ dysfunction compared to matched historical controls, a clear significant beneficial effect was seen (15).

In patients with severe COVID-19 there may be an underlying immunological mechanism of action, comparable to a mechanism we recently showed in sepsis (15), which is a hyper-inflammatory pathway associated with increased mortality. Therefore, two studies of five and 16 subjects (NCT04513470 and NCT04590053, respectively) were designed to determine the safety and efficacy of treatment with Allocetra-OTS in patients with severe/critical COVID-19.

## 2 Materials and methods

### 2.1 Study approval

The studies were approved by the local ethics committee and the Israeli Ministry of Health (Approval no. HMO–0204–20, and HMO–0770–20) and were performed according to institutional guidelines. Written informed consent was obtained in accordance with the Declaration of Helsinki from all study subjects and apoptotic cell donors.

### 2.2 Study design

The primary aim of these phase I/II studies (ClinicalTrials.gov Identifiers: NCT04513470 and NCT04590053, respectively) was to determine the safety profile of apoptotic cell infusion in subjects presenting to the Emergency Room (ER) with severe COVID-19 and respiratory dysfunction. Eligible patients with severe COVID-19 received treatment with Allocetra-OTS (Enlivex Therapeutics Ltd., Israel) apoptotic cells administered IV, followed by monitoring for adverse events (AEs) for 28 days. The secondary aims were to measure recovery from ARDS, intensive care unit (ICU) and hospital length-of-stays, and mortality.

Patients included in these studies were adult males and females aged 18–85 years, diagnosed with COVID-19, as defined below:

- Laboratory confirmation of SARS-COV2 infection by reverse-transcription polymerase chain reaction (RT-PCR) from any diagnostic sampling source.
- Patients classified as severe or critical according to National Institute of Health (NIH) severity classification (16).
- Illness with at least one of the following findings: (a) radiographic lung infiltrates, (b) arterial oxygen saturation (SpO_2_) ≤ 94% on room air, (c) requiring supplemental oxygen, with a partial pressure to fractional inspired concentration of oxygen (P/F) ratio of ≤350, ≥150.
- Exclusion criteria included: pregnancy, lactation, and woman of childbearing age who were not willing to use acceptable contraceptive measures for the entire study duration; other organ failures requiring organ support (not including mechanical ventilation), including kidney disease at stage 4 or requiring dialysis (i.e. estimated glomerular filtration rate (eGFR) < 30); malignancy; other serious systemic disease; psychosis; participation in other clinical trials or treatment with any experimental agents that may contradict this trial (e.g. biologics); co-infection with human immunodeficiency virus (HIV) or tuberculosis; known immuno-compromised state or medications known to be immunosuppressive; intubation (due to inability to sign an informed consent); partial arterial oxygen fraction (PaO_2_) to fractional inspired oxygen fraction (FiO_2_) (P/F) ratio or oxygen saturation to fractional inspired oxygen concentration (S/F) ratio <150 or change in status of eligibility manifested by a rapid decline in P/F ratio between eligibility status and actual drug delivery.

The National Early Warning Score 2 (NEWS2) (17) and 7-point ordinal scores were measured at enrollment and at each study time point. We also obtained blood samples for exploratory biological tests, including pro- and anti-inflammatory cytokines, chemokines, and growth factor levels in the serum.

Allocetra-OTS was administered as a single dose of 140×10^6^ cells/kg or as a fixed dose of 10^9^/patient, on day +1 (day 0 was time of diagnosis in the ER), following initiation of available standard-of-care (SOC) treatment for COVID-19, including remdesivir (Verklury, Gilead Sciences, Foster City, CA, USA), dexamethasone, and prophylactic enoxaparin. Interim safety analyses were performed by the Data and Safety Monitoring Board (DSMB) after 3 and 5 patients.

### 2.3 Alloctera™-OTS preparation

Enlivex Therapeutics Ltd. developed Allocetra-OTS based on the known activity of naturally occurring apoptotic cells to induce a pro-homeostatic state for both macrophages and dendritic cells (DCs) (18–22), contributing to the maintenance of peripheral homeostasis of almost all immune-triggered mechanisms in sepsis. Allocetra-OTS is composed of frozen non-matched human leukocyte antigen (HLA) mononuclear enriched leukocytes derived from a single donor via leukapheresis (Cell-Generation, Ltd. Hadassah Bio Park, Jerusalem, Israel), followed by freezing and preserving in liquid nitrogen (LN2). Following controlled thawing, cells are incubated with corticosteroids and washed to achieve an early apoptotic state. The cells are then washed again, eliminating both corticosteroid residues as well as material that may have been secreted by apoptotic cells. Once the cells are in an early apoptotic state, they are further Gamma or X-ray irradiated at 4000cGy to prevent T cell proliferation of potentially viable cells and therefore avoid the possible development of an acute graft-versus-host (GvHD)-like response and preserved at 2–8°C. Cell populations contain at least 40% early apoptotic cells in the form of a liquid suspension that is administered intravenously (IV). Following irradiation, the product is stable for up to 96 hours and kept at 2–8°C to avoid progression of apoptosis. All blood donors of Allocetra-OTS are tested for bacterial and virus infections as required for blood and plasma donation. The final product is retested for infection using pre-release Gram-stain and post-release sterility culture.

### 2.4 Potency assay

PBMC-derived monocytes/macrophages were co-cultured with Allocetra-OTS at ratios of 1:16, 1:32 and 1:64 for 1 hour. Following incubation, co-cultures were exposed to LPS and incubated for an additional 3 hours and tested by flow-cytometry for HLA-DR expression. Untreated monocytes/macrophages were used to determine basal expression levels. Monocytes/macrophages stimulated with LPS alone (without pre-incubation with Allocetra-OTS) were used as positive controls to determine upregulated expression levels in maturation response that was significantly inhibited upon adding Allocetra-OTS. This assay was based on an assay used in a previous clinical study (21) where maturation of dendritic cells was used for ApoCell potency effect.

### 2.5 Luminex® cytokine/chemokine analysis

Serum cytokine/chemokine measurements were performed as previously described (15) using the Luminex MAGPIX system (Luminex Corp, Texas, USA) and analyzed with Milliplex analysis software (Millipore MA, USA).

### 2.6 ELISA analysis

The following cytokines/chemokines were measured by sandwich ELISA kits: IL-18 (R&D), MCP-3 (R&D), TNFR1 (R&D).

### 2.7 Statistics

Comparisons between treated COVID-19 patients and healthy individuals were done using the non-parametric Mann-Whitney test. Comparisons between blood counts/cytokine levels from day 1 to day 28 for each patient were analyzed using the Wilcoxon matched-pairs signed-rank test. Means, standard deviations, medians, interquartile ranges, and ranges in each treatment group were presented, as appropriate. Statistical analysis was performed using GraphPad (Prism, San Diego, CA, USA).

## 3 Results

Following confirmation of the safety of the study in the first five patients, a follow-up study with an additional 16 patients, using the same protocol (apart from dose that was calculated 70x10^6^/kg in the first five patients and a fixed dose of 10^9^ cells per patient in the rest of the patients) was carried out. The results of the two studies together are presented here, as the same protocol was used in both.

A total of 25 patients were recruited to two consecutive studies (NCT04513470 and NCT04590053); 21/25 patients were included (median age of 53 years, 16 males (76.2%)). Patient 04 (Study NCT04513470) withdrew, patients 01-006 and 01-011 withdrew their consent, and Patient 01-010 was disqualified after recruitment due to late screen failure (Study NCT04590053). All patients received the standard of care (SOC) for COVID-19, including remdesivir (17/21, 81%), dexamethasone (21/21, 100%), and prophylactic enoxaparin (21/21, 100%). The data safety management board (DSMB) met following inclusion of 3/5 patients in the first study for a safety review and approval to continue the study, and for final review after 21 patients were enrolled. Allocetra-OTS infusion in the first five patients met the protocol for defined safety criteria at day 14 and the study proceeded to the second round of patient recruitment at the same dose, which also met the protocol-defined criteria for safety.

Patient characteristics are presented in **Table 1**.

**Table 1:** Characteristics of Patients Upon Entry to the Study.

Patient	001	002	003	005	006	01-001	01-002	01-003	01-004	01-005	01-007	01-008	01-009	01-012	01-013	01-014	01-015	02-001	02-002	02-003	03-001	All (21)
Age – years	44	53	47	59	46	57	35	61	46	47	57	42	50	66	49	65	56	37	81	66	53	Mean ± SD: 53.2 ± 10.7; Median (range): 53 (35–81)
Gender	Female	Male	Male	Female	Male	Male	Male	Male	Male	Male	Male	Male	Female	Male	Male	Male	Female	Male	Female	Male	Male	16 Males; 5 Females
Ethnic group	Arab	Arab	Jewish	Arab	Jewish	Jewish	Jewish	Jewish	Arab	Jewish	Arab	Arab	Arab	Jewish	Jewish	Jewish	Jewish	Jewish	Jewish	Jewish	Jewish	7 Arabs; 14 Jews
Weight (kg.)	80	75	108	100	100	96	125	120	80	93	95	100	70	110	120	65	75	77	80	87	96	Mean ± SD: 93 ± 16.8; Median (range): 95 (65–125)
Coexisting conditions:																						
Hypertension	no	no	no	yes	no	yes	no	yes	no	no	no	yes	yes	no	no	no	yes	no	yes	no	yes	8/21
Diabetes mellitus	no	no	no	yes	no	no	no	no	no	no	no	no	no	no	no	yes	no	no	no	no	yes	3/21
Chronic kidney disease	no	no	no	no	no	yes	no	no	no	no	yes	no	yes	no	no	no	no	no	no	no	no	3/21
Ischemic heart disease	no	no	no	no	no	no	no	no	no	no	no	no	no	no	no	no	no	no	no	no	yes	1/21
Congestive heart failure	no	no	no	no	no	no	no	no	no	no	no	no	no	no	no	no	no	no	no	no	no	0/21
Pregnancy	no	no	no	no	no	NA	NA	NA	NA	NA	NA	NA	no	NA	no	NA	no	no	no	no	NA	0/21
Overweight (BMI>25)	yes	no	yes	yes	yes	yes	yes	yes	no	yes	no	yes	no	yes	yes	no	yes	no	yes	yes	yes	15/21
Obesity (BMI >30)	yes	no	no	yes	yes	yes	yes	yes	no	yes	yes	yes	no	yes	yes	no	no	no	no	yes	yes	13/21
Chronic lung disease	no	no	no	no	no	no	no	no	no	no	no	no	yes	yes	no	no	no	no	no	yes	no	3/21
Chronic liver disease	no	no	no	no	no	no	no	no	no	no	no	no	no	no	no	no	no	no	no	no	no	0/21
Immunosuppression	no	no	no	no	no	no	no	no	no	no	no	no	no	no	no	no	no	no	no	no	no	0/21
Malignancy	no	no	no	no	no	no	no	no	no	no	no	no	no	no	no	no	no	no	no	no	no	0/21

NA, Not applicable.

### 3.1 Clinical course

Of the 21 patients, 11 were classified as having “severe” COVID-19 and 10 as “critical” COVID-19 based on the National Institute of Health (NIH) severity classification (23). Additionally, 19/21 patients had mild-to-severe (ARDS) upon hospitalization, with an initial median 7-point severity scale of 4 (range 3–4) and median NEWS2 score of 5 (range 2–8). All patients were alive at completion of 28 days of follow-up, and 19/21 (90.5%) had completely recovered. Polymerase chain reaction (PCR) testing was negative at discharge for 12/15 patients who were tested. A summary of clinical characterizations of patients included in the study is presented in **Table 2**.

**Table 2:** COVID-19 Summary of Clinical Characterizations of patients included in the study.

Patient	001	002		003	005	006	01-001	01-002	01-003	01-004	01-005	01-007		01-008	01-009	01-012	01-013			01-014	01-015	02-001	02-002		02-003	03-001	All (21)
**Covid-19 in real time PCR assay upon presentation**	Yes	Yes		Yes	Yes	Yes	Yes	Yes	Yes	Yes	Yes	Yes		Yes	Yes	Yes	Yes			Yes	Yes	Yes	Yes		Yes	Yes	21/21
NIH severity classification*	Severe	Critical		Critical	Severe	Critical	Critical	Severe	Severe	Severe	Severe	Severe		Severe	Severe	Critical	Critical			Critical	Severe	Critical	Severe		Critical	Critical	10 critical 11 severe
Current Treatment:																											
Zinc	no	no		no	no	no	no	no	no	no	no	no		no	no	no	no			no	no	no	no		no	no	0/21
Vitamin D	no	no		no	no	no	no	no	no	no	no	no		no	no	no	no			no	no	no	no		no	no	0/21
Hydroxychloroquine	no	no		no	no	no	no	no	no	no	no	no		no	no	no	no			no	no	no	no		no	no	0/21
Azithromycin	no	no		no	no	no	no	no	no	no	no	no		no	no	no	no			no	no	no	no		no	no	0/21
Lopinavir/Ritonavir	no	no		no	no	no	no	no	no	no	no	no		no	no	no	no			no	no	no	no		no	no	0/21
Convalescent plasma	no	no		no	no	no	no	no	no	no	no	no		no	no	no	no			no	no	no	yes		yes	no	2/21
Non-specific IVIG	no	no		no	no	no	no	no	no	no	no	no		no	no	no	no			no	no	no	no		no	no	0/21
Tocilizumab	no	no		no	no	no	no	no	no	no	no	no		no	no	no	no			no	no	no	no		no	no	0/21
Enoxaparin prophylaxis	yes	yes		yes	yes	yes	yes	yes	yes	yes	yes	yes		yes	yes	yes	Yes			yes	yes	yes	yes		yes	yes	21/21
Remdesivir	No	Yes		Yes	Yes	Yes	yes	yes	yes	yes	yes	yes		yes	yes	no	Yes			no	no	yes	yes		yes	yes	17/21
Favipravir	no	no		no	no	no	no	no	no	no	no	no		no	no	no	no			no	no	no	no		no	no	0/21
Dexamethasone	yes	yes		yes	yes	yes	yes	yes	yes	yes	yes	yes		yes	yes	yes	Yes			yes	yes	yes	yes		yes	yes	21/21
O_2_ nasal canula	yes	no		no	yes	no	no	yes	yes	yes	yes	yes		yes	yes	yes	no			no	yes	no	yes		no	no	12/21
Facial mask/High flow oxygen	no	yes		yes	no	yes	yes	no	no	no	no	no		no	no	no	yes			yes	no	yes	no		Yes	yes	9/21
Invasive ventilation	no	no		no	no	no	no	no	no	no	no	no		no	no	no	no			no	no	yes	no		no	no	1/21
**Lung infiltrates**	yes	yes		yes	yes	yes	yes	yes	yes	yes	yes	yes		yes	yes	yes	yes			yes	yes	yes	yes		Yes	yes	21/21
**Sat/FIO_2_ Concentration at the day of investigational product administration**	306	121		242	335	268	235	211	348	287	332	261		306	335	209	151			132	232	99	290		157	175	Mean ± SD: 240 ± 75; Median (range): 242 (99–348)
**Minimal Sat/FIO_2_ Concentration at the day of investigational product administration**	306	121		242	335	268	235	206	225	220	332	261		306	332	153	151			132	232	99	290		157	175	Mean ± SD: 228 ± 72; Median (range): 232 (99–335)
**ARDS**	Mild	Moderate		Moderate	No	Mild	Mild	Mild	Mild	Mild	Mild	Mild		Mild	No	Moderate	Moderate			Moderate	Mild	Severe	Mild		Moderate	Moderate	19/21 ARDS (11/19 mild; 8/19 moderate-severe)
**Patient’s admission day**	30/07/2020	27/08/2020		11/09/2020	16/09/2020	21/09/2020	13/10/2020	19/10/2020	22/10/2020	28/10/2020	05/11/2020	18/11/2020		24/11/2020	29/11/2020	13/12/2020	29/12/2020			02/01/2021	13/01/2021	23/11/2020	01/01/2021		10/01/2021	02/12/2020	30/07/2020 – 13/01/2021
**Day 1: investigational product administration**	04/08/2020	30/08/2020		14/09/2020	22/09/2020	23/09/2020	20/10/2020	21/10/2020	25/10/2020	29/10/2020	08/11/2020	19/11/2020		26/11/2020	02/12/2020	15/12/2020	31/12/2020			03/01/2021	18/01/2021	29/11/2020	07/01/2021		13/01/2021	07/12/2020	04/08/2020 – 18/01/2021
**Allocetra-OTS dosage (× 10^6^/kg)**	139	144		118	142	128	121	73	83	143	125	121		107	152	81	94			166	148	140	139		119	109	Mean ± SD: 123 ± 24**×** 10^6^/kg
**Total Cells per dose (× 10^9^)**	11.1	10.8		12.8	14.3	12.8	11.6	9.1	10	11.5	11.6	11.5		10.7	10.7	8.9	11.3			10.8	11.1	10.8	11.1		10.4	10.4	11 ± 1**×**10^9^ total cells/dose
**Time to Sat/O2 Concentration ≥315 (from investigational product administration, days)**	3	11		8	2	4	13	6	13	4	2	6		6	13	Did not reach Sat/O2 concentration ≥315	8			14	3	5	7		7	Did not reach Sat/O2 concentration ≥315	19/21 reached Sat/O2 Concentration ≥315; by an average of 7.1 days; median of 6 days (2-14)
**Day of dis\charge**	09/08/2020	11/09/2020		22/09/2020	24/09/2020	29/09/2020	26/10/2020	27/10/2020	29/10/2020	04/11/2020	10/11/2020	22/11/2020		29/11/2020	08/12/2020	Not discharged but end of study 11/01/2021	27/01/2021			31/01/2021	14/02/2021	29/11/2020	11/01/2021		18/01/2021	Not discharged but end of study 30/12/20	09/08/2020 – 14/02/2021
**Covid-19 in real time PCR assay upon discharge**	negative	negative		negative	negative	negative	negative	negative	negative	negative	positive	positive		positive	ND	ND	negative			negative	negative	ND	ND		ND	ND	Out of 15 that were done, 12 were negative and 3 “positive”
**Day 28 ( ± 2)**	31/08/2020	29/09/2020		11/10/2020	19/10/2020	20/10/2020	16/11/2020	16/11/2020	22/11/2020	25/11/2020	06/12/2020	16/12/2020		23/12/2020	07/01/2021	11/01/2021	27/01/2021	31/01/2021			14/02/2021	24/12/2020	03/02/2021	09/02/2021		30/12/2020	31/08/2020 – 14/02/2021
**Total hospitalization days after investigational product administration (days)**	5	12		8	2	6	6	6	4	6	2	3		3	6	28	6	9			3	4	6	7		28	Mean ± SD: 7.6 ± 7; Median (range): 6 (2–28); For discharged (19/21)- Mean: 5.5 ± 2.4 Median: 6 (2–12)
**Total hospitalization days**	10	15		11	8	8	13	8	7	7	5	4		5	9	30	8	11			8	7	11	9		30	Mean ± SD: 10.7 ± 6.8; Median (range): 8 (4–30); For discharged (19/21)- Mean: 8.6 ± 2.7 Median: 8 (4–15)
**NEWS 2 upon entry**	8	5		3	5	4	6	8	4	6	4	3		2	4	8	5	5			7	5	4	3		7	Mean ± SD: 5 ± 1.7; Median (range): 5 (2–8)
**Time to basal (before Covid 19) NEWS2 (days)**	6	13		13	6	6	7	6	7	7	6	6	6		7	Did not reach basal NEWS2	4	6			7	6	7	6		Did not reach basal NEWS2	19/21 achieved baseline (before Covid19) NEWS2 within: Mean ± SD: 6.9 ± 2.2; Median (range): 6 (4–13) days
**7 points severity scale upon entry**	4	3		3	4	4	3	4	4	4	4	4	4		4	3	4	4			4	3	4	3		3	Mean ± SD: 3.7 ± 0.5; Median (range): 4 (3–4)
**Time to basal Points in 7 points severity scale (days)**	6	13		13	6	6	27	13	13	13	4	4	5		3	Did not reach basal 7-point severity scale	6	14			7	7	7	7		Did not reach basal 7-point severity scale	19/21 returned to basal 7 points within: Mean ± SD: 9.2 ± 5.5; Median (range): 7 (3–27) days
**ICU/Death**	no	no		no	no	no	no	no	no	no	no	no	no		no	ICU	no	no			no	no	no	no		ICU	2/21 were transferred to ICU; 0/21 death by Day 28

ND, Not Detected.

### 3.2 Safety parameters

Safety was evaluated by monitoring for serious adverse events (SAEs) and adverse events (AEs). All patients survived 28 days of follow-up. Three unrelated SAEs were documented in two patients who required mechanical ventilation. No AEs related to investigational product administration were reported in 21 patients, with one AE possibly related to the investigational product administration in these studies (shivering following investigational product administration) (**Table 3**).

**Table 3:** List of Reported SAEs.

Patient	SAE reported/Study Day	Relevance to the investigational product	Resolution	Comments
01-012	20/12/2020 day 5	No relevance	Not resolved	Admission to ICU for close respiratory supervision
01-012	23/12/2020 day 8	No relevance	Not resolved	Intubation, mechanical ventilation + ECMO
03-001	13/12/2020 day 3	No relevance	Not resolved	Intubation, mechanical ventilation + ECMO

### 3.3 Preliminary efficacy results

#### 3.3.1 Morbidity and mortality

All 21 patients from both studies were alive on day 28; 19/21 had been discharged healthy from the hospital and 2/21 were in ICU on day 28. On the 7-point ordinal severity scale, the initial median score was 4 (range 3–4) and the initial median NEWS2 score was 5 (range 2–8). A complete clinical recovery and hospital discharge were achieved for 19/21 patients, as reflected by the normal ordinal score (achieved by a median 7 days) and NEWS2 score (achieved by a median of 6 days) at hospital discharge.

#### 3.3.2 Recovery from ARDS

Prevention of respiratory deterioration associated with COVID-19, particularly ARDS, was a preliminary efficacy parameter tested in this study. Out of 21 patients, there were 19 patients with ARDS, of whom 17/19 (89.5%) completely recovered. Specifically, the recovered patients reached a P/F ratio of more than 315mmHg (indicative of recovery from ARDS), after a median time of 6 days (range 2–14).

**Figure 1:**
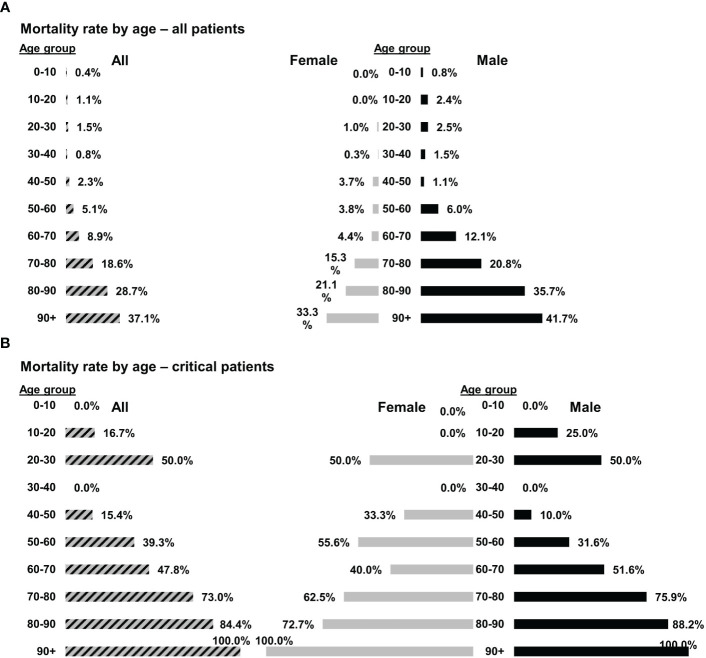
Mortality rates of COVID-19 patients at Hadassah Medical Center. The mortality rates of **(A)** all COVID-19 patients (n = 4,177) or **(B)** only critical COVID-19 patients (n = 197) that were hospitalized at the Hadassah Medical Center during the time of the studies.

Despite the severe and critical clinical conditions and ARDS in 19/21 patients, there were no mortalities within 28 days. At the same time and for the same variants (gamma, alpha, and delta) mortality at Hadassah Medical Center was 5.1% and 8.9%, for all patients aged 50–60 and 60–70, respectively. For females, the mortality rates were 3.8% and 4.4.% for females aged 50−60 and 60−70, respectively. The corresponding mortality rates among males were higher, 6.0% and 12.1% for male patients aged 50–60 and 60–70, respectively (**Figure 1A**). The mortality rates among critical COVID–19 patients (10/21 of the patients in this study, all males) hospitalized in Hadassah Medical Center during that period were even higher: 39.3% and 47.8% for critical patients aged 50–60 and 60–70, respectively; 55.6% and 40.0% for females ages 50–60 and 60–70, respectively; and 31.6% and 51.6% for males ages 50–60 and 60–70, respectively (**Figure 1B**).

#### 3.3.3 Length of stay in ICU and hospital

Since most of the patients had recovered from ARDS and COVID-19, and no mortality occurred, we were next interested to verify that these promising results were expressed in the duration of hospital stay.

Median total hospitalization after investigational product administration was 6 days (range 2–28) for all the patients (10 critical, 11 severe patients), and the average was 7.6 ± 7 days. The average time for discharge from the hospital after investigational product administration for the 19/21 patients who were discharged was 5.5 ± 2.4 days (range 2–12, median 6 days). Only 2/21 patients (01-012 and 03-001, both critical at admission) were admitted to the ICU, where they remained until the end of the study.

#### 3.3.4 Laboratory results

**Figure 2:**
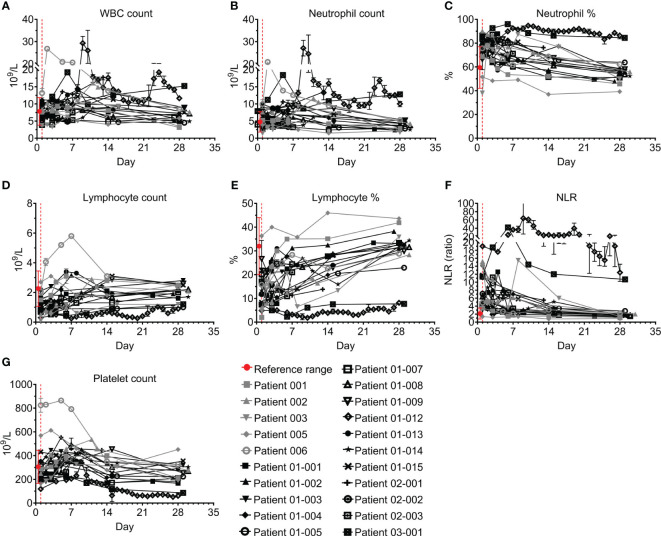
Blood counts of COVID-19 patients treated with Allocetra-OTS. Severe/critical COVID-19 patients from studies NCT04513470 (gray symbols) and NCT04590053 (black symbols) were treated with Allocetra-OTS on day 1 (red dotted line); blood samples were analyzed for **(A)** white blood cells (WBCs), **(B, C)** neutrophils, **(D, E)** lymphocytes, **(F)** neutrophil/lymphocyte ratio (NLR), and **(G)** platelet counts at the indicated timepoints. The first blood sampling for each patient was taken before IV administration of Allocetra-OTS. Data are shown as average ± range.

Laboratory evaluation included blood and serum levels, as detailed below. Complete blood count (CBC), acute phase and cardiac markers, and liver markers and enzymes of all 21 patients were documented. **Figures 2** **–** **4** and **Tables S1–S3** show their levels during the study period. White blood cell (WBC) counts were within normal limits throughout the study in 18/21 patients, and indeed were mildly decreased by 23% (IQR 8-35%) (**Figure 2A**). Neutrophil counts were above normal on admission in 7/21 patients (**Figure 2B**), and 10/21 patients presented to the ICU with an increased neutrophil percentage (**Figure 2C**). Both neutrophil count and percentage decreased significantly during the follow-up period, with median decreases of 45% and 29%, respectively between days 1 and 28 (p=0.0334 and p<0.0001 for neutrophil count and percentage, respectively, Wilcoxon matched-pairs test).

Most patients presented with lymphopenia; lymphocyte counts were below normal levels in 14/21 (**Figure 2D**) and 20/21 had a lymphocyte percentage below the normal range (**Figure 2E**). All patients except for patients 01-012 and 03-001 had a gradual increase in lymphocyte numbers and percentages, which recovered to normal levels between days 1 and 28 with median increases of 1.9- and 2.1-fold, respectively (p<0.0001, Wilcoxon matched-pairs test). In accordance with the slight neutrophilia and lymphopenia in most of the patients, 18/21 patients had a hypernormal neutrophil-to-lymphocyte ratio (NLR) upon admission to the ICU (median of 6.4, IQR: 4.5–11.3, compared to a normal range of 0.78–3.53). Except for patients 01-012 and 03-001, who had severe lymphopenia and a high NLR index throughout the study, the NLR of all the patients was significantly decreased following treatment (median decrease of 75%, IQR:29–74%) and reached normal levels by day 28 (**Figure 2F**; p<0.0001, Wilcoxon matched-pairs test). 17/21 patients had normal platelet counts during follow-up; two patients (005 and 006) had high platelet counts on admission followed by a decrease to normal levels; and patients 01-012 and 03-001 had unresolved severe thrombocytopenia (median platelet count <100×10^9^/L) (**Figure 2G**).

**Figure 3:**
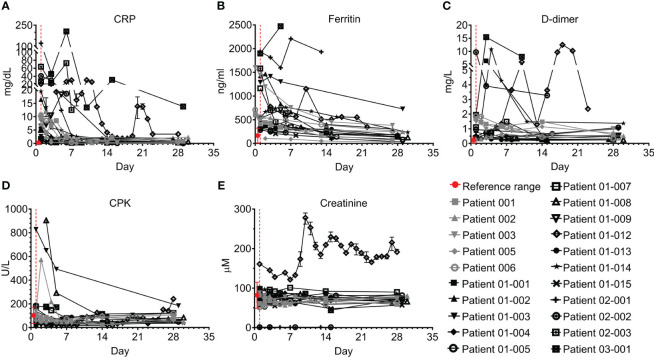
Acute phase and cardiac markers of COVID-19 patients treated with Allocetra-OTS. Severe/critical COVID-19 patients from studies NCT04513470 (gray symbols) and NCT04590053 (black symbols) were treated with Allocetra-OTS on day 1 (red dotted line). Blood samples were analyzed for **(A)** CRP, **(B)** ferritin, **(C)** D-dimer, **(D)** CPK, and **(E)** creatinine at the indicated timepoints. The first blood sampling for each patient was taken before IV administration of Allocetra-OTS. Data are shown as average ± range.

**Figure 4:**
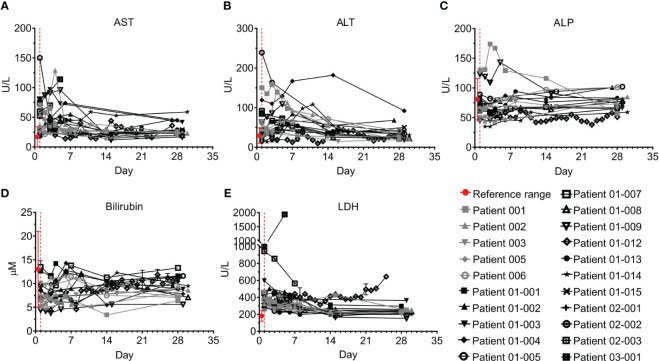
Liver markers and enzymes of COVID-19 patients treated with Allocetra-OTS. Severe/critical COVID-19 patients from studies NCT04513470 (gray symbols) and NCT04590053 (black symbols) were treated with Allocetra-OTS on day 1 (red dotted line). Blood samples were analyzed for **(A)** AST, **(B)** ALT, **(C)** alkaline phosphatase (ALP), **(D)** total bilirubin, and **(E)** LDH at the indicated timepoints. The first blood sampling for each patient was taken before IV administration of Allocetra-OTS. Data are shown as average ± range.

All patients had elevated C-reactive protein (CRP) levels and 18/21 had elevated ferritin levels; however, these acute phase markers significantly declined in parallel with resolution of inflammation (**Figures 3A, B**; p<0.0001, Wilcoxon matched-pairs test). CRP levels remained above the normal range in 10/18 patients who were tested on Day 28, especially in patients 01-012 and 03-001. Ferritin levels were within the normal range by day 28 in 14/17 patients who were tested. Most patients had normal or mildly elevated D-dimer levels, which either decreased or remained unchanged during the study. Patients 01-014, 01-012, and 03-001 had an initial increase of D-dimer. This was followed by a decrease in patient 01-14, while patients 01-012 and 03-001 maintained hyper-normal levels of D-dimer throughout the hospitalization period (**Figure 3C**). 15/19 of the tested patients had normal creatine phosphokinase (CPK) levels throughout the study; patients 01-002, 01-003, 01-008, and 02-002 initially had higher than normal CPK levels, which were gradually decreased to normal, while Patient 01-012 started with normal CPK levels and gradually developed elevated CPK (**Figure 3D**). Patient 01-012 also had sustained high creatinine levels, while all the other patients had normal creatinine levels (**Figure 3E**). As shown in **Figure 4**, most patients had abnormal levels of liver enzymes and metabolites, suggesting aberrant liver function; aspartate transaminase (AST) levels upon admission were high in 13/20 of the tested patients with a gradual decline to the normal range by day 7 and significantly decreased AST on day 28 compared to day 1 (**Figure 4A**, p=0.0434, Wilcoxon matched-pairs test). The trend of alanine transaminase (ALT) was similar to AST, with 9/19 of the tested patients having abnormally high ALT on admission followed by decreasing ALT up to day 28 in most of these patients (**Figure 4B**, p=0.0407, Wilcoxon matched-pairs test). ALP and bilirubin were within the normal range in almost all patients (**Figures 4C, D**). Lactate dehydrogenase (LDH) was high in 19/20 of the patients upon admission, and significantly decreased towards normal levels by day 14 up to day 28 in all the patients except for patient 01-012, whose LDH levels were increased by 2.3-fold (**Figure 4E**, p=0.0038, Wilcoxon matched-pairs test).

Of note, patients 01-012 and 03-001, who had markedly elevated neutrophils and CRP, did not improve as the others had and required treatment on mechanical ventilation and extracorporeal membrane oxygenation (ECMO).

Overall, for most patients, lymphocyte counts improved, and levels of acute phase reactants, CRP, ferritin, and D-dimer, decreased following treatment with Allocetra-OTS, correlating with amelioration of their clinical status. Most patients had mild elevation of liver enzymes before Allocetra-OTS administration that then resolved by day 28.

### 3.4 Exploratory endpoints, effect of Allocetra-OTS on cytokines, chemokines, and growth factors

#### 3.4.1 Pro-inflammatory cytokines

**Figure 5:**
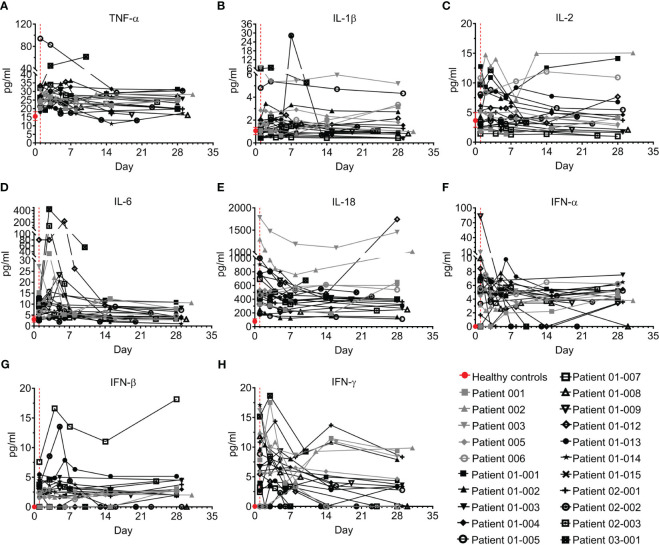
Pro-inflammatory cytokines of COVID-19 patients treated with Allocetra-OTS. Severely/critically ill COVID-19 patients from studies NCT04513470 (gray symbols) and NCT04590053 (black symbols) were treated with Allocetra-OTS on day 1 (red dotted line). Serum samples were analyzed for **(A)** TNF-α, **(B)** IL-1β, **(C)** IL-2, **(D)** IL-6, **(E)** IL-18, **(F)** IFN-α, **(G)** IFN-β, and **(H)** IFN-γ cytokine concentration at the indicated timepoints. The first blood sampling for each patient was taken before IV administration of Allocetra-OTS. Data are shown as average ± range.

Eight pro-inflammatory cytokines were tested, including TNF-α, IL-1β, IL-2, IL-6, IL-18, and interferons α, β, and γ (**Figure 5**; **Table S4**).

TNF-α has previously been reported to be elevated in the serum and plasma of COVID–19 patients compared to healthy individuals (24–26). Accordingly, most of the patients in this study had elevated TNF-α upon admission to the hospital, with median of 25.2 pg/ml (IQR: 21.9–27.2) versus a median level of 14.6 pg/ml (IQR: 13.1–18.2) in healthy individuals (p=0·0002, Mann-Whitney test). Although TNF-α levels in 10/21 of the patients (47.6%) decreased following treatment, in four patients (19%) the level was unchanged, and 6/21 patients (28.6%) had mild increases of TNF–α by day 28. Patient 03-001 had an unusual 2-fold elevation of TNF-α (**Figure 5A**).

Unlike TNF-α, the levels of IL-1β and IL-2 were not significantly different than those of healthy individuals (**Figures 5B, C**). As reported in other studies (7, 8, 27–30), IL-6 levels of the majority of the COVID–19 patients in the present study (18/21, 85.7%) were significantly higher than healthy individuals (median of 9 pg/ml and 3 pg/ml in COVID–19 patients and healthy individuals, respectively; p=0·0018, Mann-Whitney test). Following treatment, IL-6 levels had rapidly and significantly decreased by day 7 and reached normal levels at day 28 (median concentration 4.1 pg/ml, p<0.0001, Wilcoxon matched-pairs test), with a median decrease of 35% (IQR: 18%–56%). Patients 01-012, 02-003 and 03-001 had an initial increase of IL-6 until days 3–7, followed by a delayed decrease (**Figure 5D**). IL-18 was also significantly higher than normal in the COVID–19 patients (median of 490 pg/ml and 69 pg/ml in COVID–19 patients and healthy individuals, respectively; p<0.0001, Mann-Whitney test). Although 14/21 patients (66.6%) had decreased IL-18 levels following treatment (median decrease of 29% at day 28 vs day 1), the IL-18 of six patients (28.6%) was increased, including patients 001, 005, 006, 01-012, and 03-001 (**Figure 5E**). Serum concentrations of IFN-α and IFN-β were low and remained unchanged in most patients, except for patient 01-007, who had an aberrant increase in IFN-β (**Figures 5F, G**).

Unlike IFN-α and IFN-β, levels of IFN-γ in COVID–19 patients upon admission to the ICU was significantly higher than normal (median of 6.7 pg/ml and 0 pg/ml in COVID–19 patients and healthy individuals, respectively, p=0.0006, Mann-Whitney test). By day 28, most patients (15/21, 71.4%) had significantly decreased IFN-γ with resolution of the disease (**Figure 5H**, p=0.0003, Wilcoxon matched-pairs test).

#### 3.4.2 Anti-inflammatory cytokines

**Figure 6:**
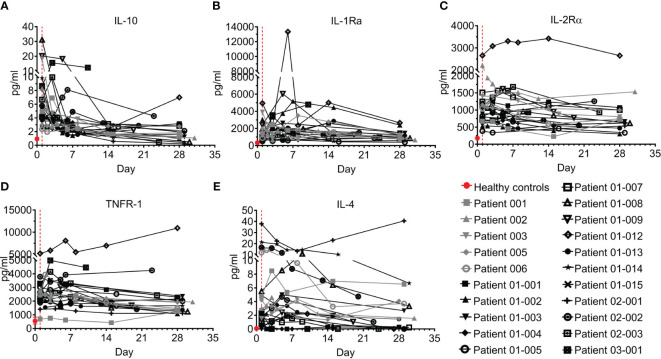
Anti-inflammatory cytokines of COVID-19 patients treated with Allocetra-OTS. Severe/critical COVID-19 patients from studies NCT04513470 (gray symbols) and NCT04590053 (black symbols) were treated with Allocetra-OTS on day 1 (red dotted line). Serum samples were analyzed for **(A)** IL-10, **(B)** IL-1Ra, **(C)** IL-2Rα, **(D)** TNFR-1, and **(E)** IL-4 cytokine concentration at the indicated timepoints. The first blood sampling for each patient was taken before IV administration of Allocetra-OTS. Data are shown as average ± range.

Interestingly, anti-inflammatory cytokines are also upregulated in COVID–19 (30, 31) in parallel with pro-inflammatory cytokines. Five anti-inflammatory cytokines were tested, including IL-10, IL-1Ra, IL-2Rα, TNFR-1, and IL-4 (**Figure 6**; **Table S5**). IL-10 was previously found to be elevated in COVID-19 patients and to be correlated with disease severity (29, 32, 33). Indeed, in the present study, IL-10 levels of COVID–19 patients on day 1 were 6.2-fold higher than levels in healthy individuals (median of 5.5 pg/ml and 0.9 pg/ml in COVID–19 patients and healthy individuals, respectively, p<0.0001, Mann-Whitney test). This was followed by rapid and substantial decline of IL-10 to normal levels in almost all the patients on days 14-to-28 (**Figure 6A**, p<0.0001, Wilcoxon matched-pairs test).

IL-1Ra, IL-2Rα, and TNFR-1 followed a similar trend. The majority of COVID–19 patients who presented with high levels of anti-inflammatory cytokines had a gradual decrease in those cytokines with resolution of the disease (**Figures 6B–D**). Unlike the majority of COVID–19 patients, patients 01-012 and p 03-001 had increasing and sustained high levels of IL-10, IL-1Ra, IL-2Rα, and TNFR-1. The pattern of IL-4 was slightly different from the other anti-inflammatory cytokines; while 20/21 patients (95%) presented with higher-than-normal IL-4 levels, most patients had decreased IL-4 levels during the follow-up period, except for patient 02-001, who manifested high levels of IL-4 throughout the study (**Figure 6E**).

Overall, the trend of the anti-inflammatory cytokines resembled that of the pro-inflammatory cytokines. Most patients had elevated IL-10, IL-1Ra, IL-2Rα, and TNFR-1, which gradually decreased as COVID-19 resolved.

#### 3.4.3 Chemokines

**Figure 7:**
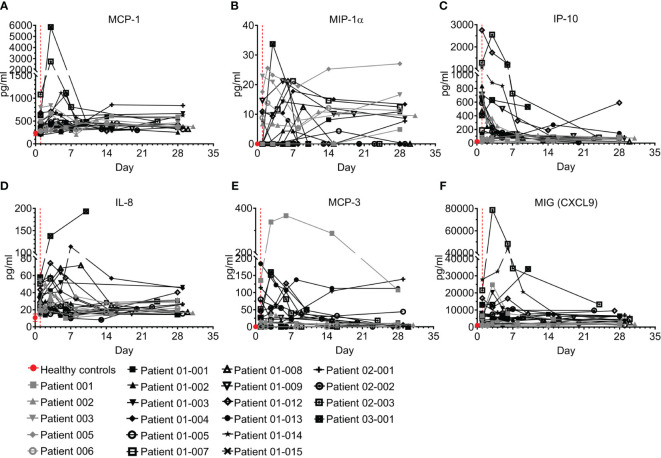
Chemokines of COVID-19 patients treated with Allocetra-OTS. Severe/critical COVID-19 patients from studies NCT04513470 (gray symbols) and NCT04590053 (black symbols) were treated with Allocetra-OTS on day 1 (red dotted line). Serum samples were analyzed for **(A)** MCP-1, **(B)** MIP-1α, **(C)** IP-10, **(D)** IL-8, **(E)** MCP-3, and **(F)** MIG (CXCL9) chemokine concentration at the indicated timepoints. The first blood sampling for each patient was taken before IV administration of Allocetra-OTS. Data are shown as average ± range.

Chemokines play pivotal roles in regulating the migration and infiltration of monocytes/macrophages and neutrophils to sites of inflammation and have been reported to be involved in COVID–19 (9, 34–36). Six chemokines were tested here, including monocyte chemoattractant protein 1 (MCP-1), macrophage inflammatory protein-1 alpha (MIP-1α), interferon gamma-induced protein 10 (IP-10), IL-8, MCP-3, and monokine induced by Gamma interferon (MIG, CXCL9) (**Figure 7**; **Table S6**). Overall, levels in most patients screened with MCP-1, MIP-1α, IL-8, and MCP-3 were above the concentration range of healthy individuals; however, IP-10 and MIG were markedly elevated in most patients upon admission to the ICU and then rapidly declined with the resolution of COVID–19. The levels of IP-10 at the end of the follow-up period remained high in patients 01-012 and 03-001, who also maintained high levels of IL-8 and MIG.

#### 3.4.4 Hematopoietic growth factors (HGFs)

**Figure 8:**
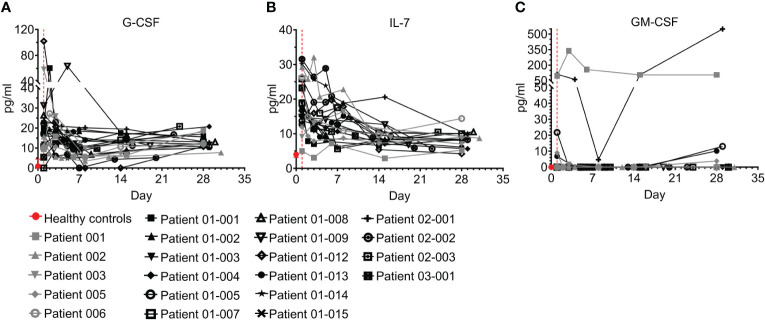
Hematopoietic growth factors (HGFs) of COVID-19 patients treated with Allocetra-OTS. Severe/critical COVID-19 patients from studies NCT04513470 (gray symbols) and NCT04590053 (black symbols) were treated with Allocetra-OTS on day 1 (red dotted line). Serum samples were analyzed for **(A)** G-CSF, **(B)** IL-7, and **(C)** GM-CSF HGF concentration at the indicated timepoints. The first blood sampling for each patient was taken before IV administration of Allocetra-OTS. Data are shown as average ± range.

An HGF is a relatively stable, secreted, or membrane-bound glycoprotein that causes immune cells to mature and/or proliferate, and also has profound effects on cell functions and behaviors. HGFs were shown to be upregulated in COVID–19 and were associated with disease severity and outcome (26, 37–39). Three growth factors were tested in the study and are presented here, including G-CSF, IL-7, and GM-CSF (**Figure 8**; **Table S7**). While GM-CSF was mostly undetected in both in healthy individuals and COVID–19 patients, ICU admission levels of G-CSF and IL-7 were significantly higher in COVID–19 patients than in healthy individuals (**Figures 8A, B**; p=0.0002 and p<0.0001 for G-CSF and IL-7, respectively). Following treatment, both HGFs were significantly decreased by day 7 and at day 28 were within or slightly above the normal range (**Figures 8A, B**; p=0.0446 and p<0.0001, for G-CSF and IL-7, respectively, Wilcoxon matched-pairs test). Overall, the trend of G-CSF and IL-7 HGFs resembled that of most of the cytokines and chemokines measured.

### 3.5 Summary

In summary, most patients had elevated pro-, and anti-inflammatory cytokines, chemokines, and HGFs upon entering the study. Following treatment with Allocetra–OTS, most of these inflammatory immune-modulators, including IL-6, IFN-γ, IL-10, TNFR1, IP-10, MCP-3, and IL-7, gradually decreased to normal levels with resolution of COVID-19.

## 4 Discussion

Apoptotic cells (Allocetra–OTS) were given to 21 COVID–19 patients (earlier strains; G, alpha and delta), 11 with severe and 10 with critical illness. 19/21 patients had significant ARDS and all patients received supplemental oxygen via high-flow nasal cannula. Overall, the treatment with Allocetra-OTS was safe. There were only three serious adverse events (SAEs) and all were documented as “unrelated” to investigational product. Allocetra-OTS was also well tolerated when given in conjunction with standard therapy (remdesivir, enoxaparin, and dexamethasone). 19/21 of subjects showed early recovery, with 5.5 days on average until discharge; only 2/21 patients were still hospitalized by day 28 (end of study). 19 patients had mild-to-severe ARDS, and 17/19 (89.5%) completely recovered within a few days. For comparison, at the same time and for the same variants (G, alpha and delta), the mortality rates among critical COVID–19 patients (10/21 of the patients in this study) that were hospitalized in Hadassah Medical Center at that time period were much higher; 39.3% and 47.8%, for critical patients aged 50–60 and 60–70 years, respectively; females 55.6% and 40.0%, for ages 50–60 and 60–70, respectively; and males 31.6% and 51.6% for ages 50–60 and 60–70, respectively (**Figure 1B**). Although there was no control group in this study, the concomitant mortality rates with these variants, gender, severity, and ARDS in our institution were between 15%−40%.

Similar to patients with other severe inflammatory conditions, such as sepsis (11, 40, 41), most of the COVID–19 patients in this study were admitted to the ICU with high levels of CRP, lymphopenia, an elevated neutrophil count, and high NLR. Cytokine/chemokine inflammatory markers resolved following treatment with Allocetra–OTS, a pattern similar to the trends that we observed after treatment of patients with mild-to-moderate sepsis with Allocetra-OTS (15).

Immunological dysregulation that is manifested by a cytokine storm has emerged as a hallmark of COVID–19-related ARDS (8, 9). Accordingly, chemokines and pro- as well as anti-inflammatory cytokines were present in high concentrations in the serum of the COVID-19 patients in this study. Following treatment with Allocetra-OTS there was rapid reduction of TNF-α and IL-6, both independent predictors of disease severity and mortality (25, 29). The resolution of cytokine storm was also demonstrated by a substantial decrease in the levels of IFN-γ, IL-10, TNFR-1, and IP-10, all of which are involved in the imbalanced and harmful immune-response typical of severe COVID-19 patients (29, 32, 35, 38). Of note, only 2/21 patients did not experience resolution of the cytokine storm; these two patients were critical patients with a NEWS2 score higher than 7 at admission. Both were hospitalized in the intensive care unit (ICU) and progressed to mechanical ventilation and then extracorporeal membrane oxygenation (ECMO) within a week from the beginning of the study. This observation may suggest that recovery is very much related to the resolution of cytokine storms.

In this study, patterns seen with a resolution of the cytokine storm in patients with severe COVID-19 following treatment with Allocetra-OTS shared some striking similarities with patterns in a previous Phase 1b study that we conducted to determine the safety profile of Allocetra-OTS treatment in patients with mild-moderate sepsis (15). Similarities included resolution of lymphopenia and neutrophilia and a rapid decrease in the serum concentrations of CRP, TNF-α, IL-6, IFN-γ, IL-10, TNFR-1, and IP-10 following treatment with Allocetra-OTS.

Elevations in these common disease markers and their resolution following treatment with Allocetra-OTS suggests two main insights: (i) severe COVID–19 and sepsis possibly share common pathogenesis of cytokine triggering mechanisms; (ii) these mechanisms may involve activated macrophages, which are major initiators and inducers of cytokine storms (35) and which are the target of Allocetra-OTS apoptotic cells. Indeed, in both sepsis and severe COVID-19, pathogen-related signals trigger the activation of tissue macrophages, which in turn induce an initial hyper-inflammatory response characterized by the release of pro-inflammatory cytokines like TNF and IL-6 (42–45). This includes the release of chemokines and the subsequent recruitment and activation of the adaptive immune system. This prolonged activation also involves the secretion of anti-inflammatory cytokines such as IL-10, which can lead to terminal immune-suppression (43, 46).

Therefore, Allocetra-OTS may offer a holistic therapeutic approach for those disease processes that involve a severely imbalanced immune-response that cannot be resolved using monotherapies.

These results suggest that immune modulation of cytokine storm with Allocetra may offer a mode of treatment for critically ill patients with ARDS; however, to verify that a controlled study is needed.

## Data availability statement

The raw data supporting the conclusions of this article will be made available by the authors, without undue reservation.

## Ethics statement

The studies involving human participants were reviewed and approved by the local ethics committee and the Israeli Ministry of Health. The patients/participants provided their written informed consent to participate in this study.

## Author contributions

PH designed the research studies, treated the patients, and wrote the manuscript. AA designed the research studies, and wrote the manuscript. AhN and SM treated the patients and wrote the manuscript. KAJ, NM and AkN treated the patients. LB designed the research studies and treated the patients. OH helped in the design of the study. YS designed the research studies, conducted the experiments, acquired and analyzed the data, and provided reagents. BR acquired and analyzed the data, and wrote the manuscript. DM designed the research studies, acquired and analyzed the data, and wrote the manuscript. All authors contributed to the article and approved the submitted version.
