# Supplementary material for: Apoptotic cells for treatment of acute respiratory distress syndrome associated with COVID-19
Source: Front Immunol. 2023 Aug 2;14:1242551. doi: 10.3389/fimmu.2023.1242551 (PMC10433372; doi:10.3389/fimmu.2023.1242551)
Supplement: Supplementary file 1 [file Table_1.docx]

Supplementary Material

Apoptotic cells for treatment of acute respiratory distress syndrome associated with COVID-19

**Peter Vernon van Heerden^1^, Avraham Abutbul^2^, Ahmad Naama^3^, Shlomo Maayan^4^, Nassar Makram^4^, Akiva Nachshon^1^, Kamal abu Jabal^5^, Oren Hershkovitz^6^, Lior Binder^6^, Yehudit Shabat^6^, Barak Reicher^6^, and Dror Mevorach^7,8*^**

*** Correspondence:** Prof. Dror Mevorach: mevorachd@hadassah.org.il

**Supplemental Tables S1–S7**

# Supplementary Tables

**Table S1. Blood counts of COVID-19 patients treated with Allocetra-OTS on Day 1 and Day 28**

|  | **WBC (10^9^/L)** | | | **Neutrophils (%)** | | | **Neutrophils (10^9^/L)** | | | **Lymphocytes (%)** | | |
| --- | --- | --- | --- | --- | --- | --- | --- | --- | --- | --- | --- | --- |
|  | **Day 1** | **Day 28** | **% change** | **Day 1** | **Day 28** | **% change** | **Day 1** | **Day 28** | **% change** | **Day 1** | **Day 28** | **% change** |
| **Patient 001** | 7.6 | 3.1 | 41% | 68.6 | 45.7 | 67% | 5.2 | 2.8 | 54% | 2 | 41.9 | >1000% |
| **Patient 002** | 8.4 | 7.6 | 90% | 90.2 | 55.8 | 62% | 7.6 | 4.2 | 55% | 5.9 | 28.4 | 481% |
| **Patient 003** | 3.7 | 3.4 | 92% | 38 | 50 | 132% | 1.4 | 1.7 | 121% | 4.7 | 35.9 | 764% |
| **Patient 005** | 7.5 | 5.8 | 77% | 51.6 | 39.2 | 76% | 3.9 | 2.2 | 56% | 36.3 | 43.6 | 120% |
| **Patient 006** | 14.5 | 8.5 | 59% | 77.2 | 62.3 | 81% | 11.2 | 5.3 | 47% | 15.9 | 28.8 | 181% |
| **Patient 01-001** | 6.8 | 4.9 | 72% | 75.5 | 51.8 | 69% | 5.1 | 2.5 | 49% | 16.4 | 33.1 | 202% |
| **Patient 01-002** | 6.1 | 5.1 | 84% | 74.3 | 47.8 | 64% | 4.5 | 2.4 | 53% | 17.5 | 38.2 | 218% |
| **Patient 01-003** | 9.7 | 8.9 | 92% | 66.3 | 56.8 | 86% | 6.4 | 5 | 78% | 21.9 | 32 | 146% |
| **Patient 01-004** | 11.2 | 7.3 | 65% | 88.3 | 58.4 | 66% | 9.9 | 4.3 | 43% | 7 | 30.5 | 436% |
| **Patient 01-005** | 9.6 | 4.5 | 47% | 82.9 | 63.7 | 77% | 7.9 | 2.8 | 35% | 7.6 | 22.9 | 301% |
| **Patient 01-007** | 3.9 | 6 | 154% | 73.9 | 51 | 69% | 2.9 | 3 | 103% | 18.1 | 33.4 | 185% |
| **Patient 01-008** | 7.2 | 7.2 | 100% | 80.5 | 53.7 | 67% | 5.8 | 3.8 | 66% | 12.4 | 31.6 | 255% |
| **Patient 01-009** | 4.4 | 8.9 | 202% | 75.5 | 58 | 77% | 3.3 | 5.2 | 158% | 18.9 | 29.9 | 158% |
| **Patient 01-012** | 6.5 | 12.4 | 190% | 88.2 | 86. 8 | 98% | 5.7 | 10.7 | 188% | 4.9 | 7.7 | 156% |
| **Patient 01-013** | 10.35 | 6.4 | 62% | 76.35 | 55.2 | 72% | 7.9 | 3.5 | 44% | 16.7 | 32.8 | 196% |
| **Patient 01-014** | 7.3 | 4.9 | 67% | 80.3 | 52.2 | 65% | 5.9 | 2.5 | 42% | 13.3 | 34.4 | 259% |
| **Patient 01-015** | 10.35 | 7.6 | 73% | 86.95 | 56.1 | 65% | 8.95 | 4.2 | 47% | 8.35 | 32.8 | 393% |
| **Patient 02-001** | 10.79 | N/A | N/A | 72.2 | N/A | N/A | 7.8 | N/A | N/A | 16.8 | N/A | N/A |
| **Patient 02-002** | 6.03 | N/A | N/A | 82.2 | N/A | N/A | 4.96 | N/A | N/A | 11.8 | N/A | N/A |
| **Patient 02-003** | 5.21 | N/A | N/A | 76.4 | N/A | N/A | 3.98 | N/A | N/A | 17.1 | N/A | N/A |
| **Patient 03-001** | 9.3 | 15.2 | 163% | 85 | 84.4 | 99% | 7.89 | 12.8 | 162% | 7.4 | 7.7 | 104% |
| **Patient median (N=)** | 7.5 (21) | 6.8 (18) | 77% (18) | 76.4 (21) | 55.2 (18) | 69% (18) | 5.8 (21) | 3.7 (18) | 54% (18) | 13.3 (21) | 32.4 (18) | 210% (18) |
| **Patient IQR** | 6.1-9.7 | 5.0-8.3 | 65%-92% | 73.9-82.9 | 51.0-58.0 | 66%-77% | 4.5-7.9 | 2.6-4.8 | 47%-78% | 7.4-17.1 | 29.1-34.2 | 175%-324% |
| **Normal range** | 3.79-11.84 | | | 42-77 | | | 1.78-7.73 | | | 22-44 | | |
| **P value** | 0.2291 | | | **<0.0001** | | | **0.0334** | | | **<0.0001** | | |
|  | **Lymphocytes (10^9^/L)** | | | **NLR** | | | **Platelets (10^9^/L)** | | |  |  |  |
|  | **Day 1** | **Day 28** | **% change** | **Day 1** | **Day 28** | **% change** | **Day 1** | **Day 28** | **% change** |  |  |  |
| **Patient 001** | 1.5 | 2.5 | 167% | 3.5 | 1.1 | 32% | 288 | 210 | 73% |  |  |  |
| **Patient 002** | 0.5 | 2.1 | 420% | 15.2 | 2 | 13% | 250 | 275 | 110% |  |  |  |
| **Patient 003** | 0.1 | 1.2 | >1000% | 14 | 1.4 | 10% | 190 | 122% | 122% |  |  |  |
| **Patient 005** | 2.7 | 2.5 | 93% | 1.4 | 0.9 | 61% | 452 | 79% | 79% |  |  |  |
| **Patient 006** | 2.3 | 2.4 | 104% | 4.9 | 2.2 | 45% | 325 | 37% | 37% |  |  |  |
| **Patient 01-001** | 1.1 | 1.6 | 145% | 4.6 | 1.6 | 34% | 166 | 69% | 69% |  |  |  |
| **Patient 01-002** | 1 | 1.9 | 190% | 4.5 | 1.3 | 28% | 213 | 92% | 92% |  |  |  |
| **Patient 01-003** | 2.1 | 2.8 | 133% | 3 | 1.8 | 59% | 319 | 93% | 93% |  |  |  |
| **Patient 01-004** | 0.7 | 2.2 | 314% | 14.1 | 2 | 14% | 177 | 50% | 50% |  |  |  |
| **Patient 01-005** | 0.7 | 1 | 143% | 11.3 | 2.8 | 25% | 223 | 80% | 80% |  |  |  |
| **Patient 01-007** | 0.7 | 2 | 286% | 4.1 | 1.5 | 36% | 210 | 60% | 60% |  |  |  |
| **Patient 01-008** | 0.9 | 2.2 | 244% | 6.4 | 1.7 | 27% | 269 | 131% | 131% |  |  |  |
| **Patient 01-009** | 0.8 | 2.6 | 325% | 4.1 | 3 | 73% | 253 | 156% | 156% |  |  |  |
| **Patient 01-012** | 0.3 | 0.9 | 294% | 19 | 12.1 | 64% | 59 | 49% | 49% |  |  |  |
| **Patient 01-013** | 1.7 | 2.1 | 124% | 6.5 | 1.7 | 25% | 292 | 104% | 104% |  |  |  |
| **Patient 01-014** | 0.9 | 1.7 | 189% | 6.6 | 1.5 | 22% | 304 | 90% | 90% |  |  |  |
| **Patient 01-015** | 0.8 | 2.5 | 313% | 11.2 | 1.7 | 15% | 355 | 82% | 82% |  |  |  |
| **Patient 02-001** | 1.56 | N/A | N/A | 5 | N/A | N/A | 286 | N/A | N/A |  |  |  |
| **Patient 02-002** | 0.7 | N/A | N/A | 7.1 | N/A | N/A | 215 | N/A | N/A |  |  |  |
| **Patient 02-003** | 0.89 | N/A | N/A | 4.5 | N/A | N/A | 192 | N/A | N/A |  |  |  |
| **Patient 03-001** | 0.69 | 1.2 | 174% | 11.4 | 10.7 | 94% | 229 | 86 | 38% |  |  |  |
| **Patient median (N=)** | 0.9 (21) | 2.1 (18) | 190% (18) | 6.4 (21) | 1.7 (18) | 30% (18) | 280.0 (21) | 238.0 (18) | 82% (18) |  |  |  |
| **Patient IQR** | 0.7-1.5 | 1.6-2.5 | 141%-299% | 4.5-11.3 | 1.5-2.2 | 23%-55% | 215-343 | 195-301 | 69%-104% |  |  |  |
| **Normal range** | 1.07-3.45 | | | 0.78-3.53 | | | 166-445 | | |  |  |  |
| **P value** | **<0.0001** | | | **<0.0001** | | | **0.0203** | | |  |  |  |

**P value of a Wilcoxon matched-pairs signed rank test, comparing between the median values of each patient’s Day 1 and Day 28.**

**Table S2. Acute phase and cardiac markers of COVID-19 patients treated with Allocetra-OTS on Day 1 and Day 28**

|  | **CRP (mg/dL)** | | | **Ferritin (ng/ml)** | | | **D-dimer (mg/L)** | | | **CPK (U/L)** | | |
| --- | --- | --- | --- | --- | --- | --- | --- | --- | --- | --- | --- | --- |
|  | **Day 1** | **Day 28** | **% change** | **Day 1** | **Day 28** | **% change** | **Day 1** | **Day 28** | **% change** | **Day 1** | **Day 28** | **% change** |
| **Patient 001** | 9.41 | 0.79 | 8% | 605 | 70 | 12% | 1.68 | 0.59 | 35% | 106 | 75 | 0.71 |
| **Patient 002** | 7.31 | 2.35 | 32% | 524.5 | 246 | 47% | 0.31 | 0.51 | 165% | 136 | 50 | 37% |
| **Patient 003** | 10.71 | 0.4 | 4% | 1600 | 382 | 24% | 1.49 | 1.01 | 68% | 138 | 56 | 41% |
| **Patient 005** | N/A | 0 | N/A | 104 | 26 | 25% | 1.28 | 0.7 | 55% | 58 | 106 | 183% |
| **Patient 006** | 10.16 | 0.68 | 7% | 709 | 264 | 37% | 1.87 | 0.32 | 17% | 101 | 117 | 116% |
| **Patient 01-001** | 1.79 | 0.43 | 24% | 373 | 133 | 36% | 6.4 | 0.45 | 7% | 21 | 59 | 281% |
| **Patient 01-002** | 16.43 | 1.89 | 12% | 1426 | 303 | 21% | 0.52 | 0.25 | 48% | 134 | 78 | 58% |
| **Patient 01-003** | 2.66 | 0.64 | 24% | 1289 | 728 | 56% | 0.29 | 1 | 345% | 829 | 184 | 22% |
| **Patient 01-004** | 0.82 | 1.2 | 146% | 487 | 189 | 39% | 0.42 | 0.5 | 119% | 86 | 63 | 73% |
| **Patient 01-005** | 0.92 | 0 | 0% | 324 | 81 | 25% | 0.94 | 1.09 | 116% | 43 | 48 | 112% |
| **Patient 01-007** | 1.65 | 0.35 | 21% | 1130 | 145 | 13% | 0.36 | 0.26 | 72% | 187 | 122 | 65% |
| **Patient 01-008** | 5.07 | 0.67 | 13% | 335 | 125 | 37% | 0.34 | 0.24 | 71% | 905 | 84 | 9% |
| **Patient 01-009** | 9.27 | 0.35 | 4% | 533 | 31 | 6% | 0.36 | 0.54 | 150% | 23 | 15 | 65% |
| **Patient 01-012** | 20.83 | 3.48 | 17% | 547 | 347 | 63% | 0.5 | 2.36 | 472% | 53 | 241 | 455% |
| **Patient 01-013** | 4.92 | 0.3 | 6% | 347 | 173 | 50% | 0.32 | 0.2 | 63% | 159 | 122 | 77% |
| **Patient 01-014** | 19.52 | 0.07 | 0% | 631 | 234 | 37% | 2.53 | 1.37 | 54% | 46 | 37 | 80% |
| **Patient 01-015** | 6.52 | 1.04 | 16% | 299 | 126 | 42% | 0.99 | 0.49 | 49% | 62.5 | 51 | 82% |
| **Patient 02-001** | 123 | N/A | N/A | 1935 | N/A | N/A | 0.77 | N/A | N/A | 77 | N/A | N/A |
| **Patient 02-002** | 40.9 | N/A | N/A | 284 | N/A | N/A | 9.712 | N/A | N/A | 186 | N/A | N/A |
| **Patient 02-003** | 63.8 | N/A | N/A | 1581 | N/A | N/A | 1.112 | N/A | N/A | N/A | N/A | N/A |
| **Patient 03-001** | 27.22 | 13.8 | 51% | 1898 | N/A | N/A | 1.95 | N/A | N/A | N/A | N/A | N/A |
| **Patient median (N=)** | 9.3 (20) | 0.7 (18) | 13% (17) | 547.0 (21) | 173.0 (17) | 37% (17) | 0.9 (21) | 0.5 (17) | 68% (17) | 101.0 (19) | 75.0 (17) | 73% (17) |
| **Patient IQR** | 4.4-19.8 | 0.4-1.2 | 6%-22% | 347-1289 | 125-264 | 24%-42% | 0.4-1.7 | 0.3-1.0 | 49%-119% | 56-149 | 51-117 | 58%-112% |
| **Normal range** | 0-0.5 | | | 10-322 | | | 0-0.44 | | | 34-171 | | |
| **P value** | **<0.0001** | | | **<0.0001** | | | 0.1704 | | | 0.0887 | | |
| **Creatinine (μM)** | | |  |  |  |  |  |  |  |  |  |  |
|  | **Day 1** | **Day 28** | **% change** |  |  |  |  |  |  |  |  |  |
| **Patient 001** | 61 | 59 | 97% |  |  |  |  |  |  |  |  |  |
| **Patient 002** | 82 | 81 | 99% |  |  |  |  |  |  |  |  |  |
| **Patient 003** | 88 | 75 | 0.85 |  |  |  |  |  |  |  |  |  |
| **Patient 005** | 82 | 75 | 91% |  |  |  |  |  |  |  |  |  |
| **Patient 006** | 54 | 65 | 120% |  |  |  |  |  |  |  |  |  |
| **Patient 01-001** | 67 | 69 | 103% |  |  |  |  |  |  |  |  |  |
| **Patient 01-002** | 68 | 63 | 93% |  |  |  |  |  |  |  |  |  |
| **Patient 01-003** | 77 | 76 | 99% |  |  |  |  |  |  |  |  |  |
| **Patient 01-004** | 81 | 84 | 104% |  |  |  |  |  |  |  |  |  |
| **Patient 01-005** | 75 | 82 | 109% |  |  |  |  |  |  |  |  |  |
| **Patient 01-007** | 102 | 90 | 88% |  |  |  |  |  |  |  |  |  |
| **Patient 01-008** | 98 | 66 | 67% |  |  |  |  |  |  |  |  |  |
| **Patient 01-009** | 93 | 66 | 71% |  |  |  |  |  |  |  |  |  |
| **Patient 01-012** | 161 | 198 | 123% |  |  |  |  |  |  |  |  |  |
| **Patient 01-013** | 60 | 72 | 120% |  |  |  |  |  |  |  |  |  |
| **Patient 01-014** | 100 | 71 | 71% |  |  |  |  |  |  |  |  |  |
| **Patient 01-015** | 56 | 57 | 102% |  |  |  |  |  |  |  |  |  |
| **Patient 02-001** | 0.71 | N/A | N/A |  |  |  |  |  |  |  |  |  |
| **Patient 02-002** | 1.26 | N/A | N/A |  |  |  |  |  |  |  |  |  |
| **Patient 02-003** | 83 | N/A | N/A |  |  |  |  |  |  |  |  |  |
| **Patient 03-001** | 69.85 | 74 | 106% |  |  |  |  |  |  |  |  |  |
| **Patient median N=)** | 77.0 (21) | 73.0 (18) | 99% (18) |  |  |  |  |  |  |  |  |  |
| **Patient IQR** | 61.0-88.0 | 66.0-79.8 | 88%-104% |  |  |  |  |  |  |  |  |  |
| **Normal range** | 49-115 | | |  |  |  |  |  |  |  |  |  |
| **P value** | 0.5295 | | |  |  |  |  |  |  |  |  |  |

**P value of a Wilcoxon matched-pairs signed rank test, comparing between the median values of each patient’s Day 1 and Day 28.**

**Table S3. Liver markers and enzymes of COVID-19 patients treated with Allocetra-OTS on Day 1 and Day 28**

| **AST (U/L)** | | | | | | **ALT (U/L)** | | | | | | **ALP (U/L)** | | | | | | **Bilirubin (μM)** | | | | | |  |  |
| --- | --- | --- | --- | --- | --- | --- | --- | --- | --- | --- | --- | --- | --- | --- | --- | --- | --- | --- | --- | --- | --- | --- | --- | --- | --- |
|  | | **Day 1** | | **Day 28** | | **% change** | | **Day 1** | | **Day 28** | | **% change** | | **Day 1** | | **Day 28** | | **% change** | | **Day 1** | | **Day 28** | | **% change** | |
| **Patient 001** | | 71 | | 26 | | 37% | | 150 | | 19 | | 13% | | 130 | | 57 | | 44% | | 5.2 | | 6.4 | | 1.23 | |
| **Patient 002** | | 54 | | 23 | | 43% | | 45 | | 21 | | 47% | | 47 | | 85 | | 181% | | 6.5 | | 8.6 | | 132% | |
| **Patient 003** | | 24 | | 19 | | 79% | | 14 | | 13 | | 93% | | 50 | | 66 | | 132% | | 10.1 | | 9.1 | | 90% | |
| **Patient 005** | | 35 | | 26 | | 74% | | 58 | | 27 | | 47% | | 67 | | 73 | | 109% | | 7.6 | | 7.3 | | 96% | |
| **Patient 006** | | 31 | | 24 | | 77% | | 64 | | 41 | | 64% | | 65 | | 101 | | 155% | | 6.9 | | 7.1 | | 103% | |
| **Patient 01-001** | | 19 | | 23 | | 121% | | 25 | | 24 | | 96% | | 61 | | 65 | | 107% | | 12.6 | | 11.6 | | 92% | |
| **Patient 01-002** | | 30 | | 46 | | 153% | | 44 | | 68 | | 155% | | 75 | | 101 | | 135% | | 9 | | 9.6 | | 107% | |
| **Patient 01-003** | | 80 | | 42 | | 53% | | 44 | | 38 | | 86% | | 48 | | 66 | | 138% | | 8.3 | | 8.8 | | 106% | |
| **Patient 01-004** | | 73 | | 45 | | 62% | | 119 | | 92 | | 77% | | 84 | | 82 | | 98% | | 9.9 | | 10.5 | | 106% | |
| **Patient 01-005** | | 150 | | 27 | | 18% | | 239 | | 34 | | 14% | | 89 | | 102 | | 115% | | 9.7 | | 11.6 | | 120% | |
| **Patient 01-007** | | 51 | | 23 | | 45% | | 87 | | 22 | | 25% | | 74 | | 67 | | 91% | | 12.2 | | 13.3 | | 109% | |
| **Patient 01-008** | | 37 | | 20 | | 54% | | 37 | | 22 | | 59% | | 71 | | 78 | | 110% | | 5.5 | | 7.1 | | 129% | |
| **Patient 01-009** | | 80 | | 17 | | 21% | | 63 | | 20 | | 32% | | 123 | | 73 | | 59% | | 4.4 | | 5.6 | | 127% | |
| **Patient 01-012** | | 20 | | 36 | | 180% | | 15 | | 25.25 | | 168% | | 46 | | 51.5 | | 112% | | 7 | | 10.8 | | 154% | |
| **Patient 01-013** | | 18 | | 29 | | 161% | | 27.5 | | 41 | | 149% | | 80.5 | | 83 | | 103% | | 5.2 | | 8.9 | | 171% | |
| **Patient 01-014** | | 38 | | 59 | | 155% | | 19 | | 31 | | 163% | | 38 | | 59 | | 155% | | 7.3 | | 9.5 | | 130% | |
| **Patient 01-015** | | 26.5 | | 29 | | 109% | | 34 | | 50 | | 147% | | 70 | | 81 | | 116% | | 8.5 | | 7.8 | | 92% | |
| **Patient 02-001** | | 52 | | N/A | | N/A | | N/A | | N/A | | N/A | | 50 | | N/A | | N/A | | N/A | | N/A | | N/A | |
| **Patient 02-002** | | N/A | | N/A | | N/A | | N/A | | N/A | | N/A | | N/A | | N/A | | N/A | | N/A | | N/A | | N/A | |
| **Patient 02-003** | | 60 | | N/A | | N/A | | 92 | | N/A | | N/A | | N/A | | N/A | | N/A | | N/A | | N/A | | N/A | |
| **Patient 03-001** | | 74 | | N/A | | N/A | | 85 | | N/A | | N/A | | N/A | | N/A | | N/A | | N/A | | N/A | | N/A | |
| **Patient median (N=)** | | 44.5 (20) | | 26.0 (17) | | 74% (17) | | 45.0 (19) | | 27.0 (17) | | 77% (17) | | 68.5 (18) | | 73.0 (17) | | 112% (17) | | 7.6 (17) | | 8.9 (17) | | 109% (17) | |
| **Patient IQR** | | 29.1-71.5 | | 23.0-36.0 | | 45%-121% | | 30.8-86.0 | | 22.0-41.0 | | 47%-147% | | 50.0-79.1 | | 66.0-83.0 | | 103%-135% | | 6.5-9.7 | | 7.3-10.5 | | 103%-129% | |
| **Normal range** | | 0-34 | | | | | | 10-49 | | | | | | 46-116 | | | | | | 5-21 | | | | | |
| **P value** | | **0.0434** | | | | | | **0.0407** | | | | | | 0.0909 | | | | | | **0.0089** | | | | | |
|  | | **LDH (U/L)** | | | | | |  | |  | |  | |  | |  | |  | |  | |  | |  | |
| **Day 1** | | **Day 28** | | **% change** | |  | |  | |  | |  | |  | |  | |  | |  | |  | |  |  |
| **Patient 001** | | 465 | | 251 | | 54% | |  | |  | |  | |  | |  | |  | |  | |  | |  | |
| **Patient 002** | | 403 | | 255 | | 63% | |  | |  | |  | |  | |  | |  | |  | |  | |  | |
| **Patient 003** | | 240 | | 202 | | 0.84 | |  | |  | |  | |  | |  | |  | |  | |  | |  | |
| **Patient 005** | | 314 | | 226 | | 72% | |  | |  | |  | |  | |  | |  | |  | |  | |  | |
| **Patient 006** | | 393 | | 226 | | 58% | |  | |  | |  | |  | |  | |  | |  | |  | |  | |
| **Patient 01-001** | | 294 | | 297 | | 101% | |  | |  | |  | |  | |  | |  | |  | |  | |  | |
| **Patient 01-002** | | 396 | | 237 | | 60% | |  | |  | |  | |  | |  | |  | |  | |  | |  | |
| **Patient 01-003** | | 598 | | 363 | | 61% | |  | |  | |  | |  | |  | |  | |  | |  | |  | |
| **Patient 01-004** | | 329 | | 258 | | 78% | |  | |  | |  | |  | |  | |  | |  | |  | |  | |
| **Patient 01-005** | | 369 | | 196 | | 53% | |  | |  | |  | |  | |  | |  | |  | |  | |  | |
| **Patient 01-007** | | 434 | | 230 | | 53% | |  | |  | |  | |  | |  | |  | |  | |  | |  | |
| **Patient 01-008** | | 295 | | 234 | | 79% | |  | |  | |  | |  | |  | |  | |  | |  | |  | |
| **Patient 01-009** | | 301 | | 152 | | 50% | |  | |  | |  | |  | |  | |  | |  | |  | |  | |
| **Patient 01-012** | | 282 | | 645 | | 229% | |  | |  | |  | |  | |  | |  | |  | |  | |  | |
| **Patient 01-013** | | 285 | | 249 | | 87% | |  | |  | |  | |  | |  | |  | |  | |  | |  | |
| **Patient 01-014** | | 392 | | 201 | | 51% | |  | |  | |  | |  | |  | |  | |  | |  | |  | |
| **Patient 01-015** | | 268.5 | | 219 | | 82% | |  | |  | |  | |  | |  | |  | |  | |  | |  | |
| **Patient 02-001** | | N/A | | N/A | | N/A | |  | |  | |  | |  | |  | |  | |  | |  | |  | |
| **Patient 02-002** | | 435 | | N/A | | N/A | |  | |  | |  | |  | |  | |  | |  | |  | |  | |
| **Patient 02-003** | | 945 | | N/A | | N/A | |  | |  | |  | |  | |  | |  | |  | |  | |  | |
| **Patient 03-001** | | 997 | | N/A | | N/A | |  | |  | |  | |  | |  | |  | |  | |  | |  | |
| **Patient median (N=)** | | 381 (20) | | 234 (17) | | 63% (17) | |  | |  | |  | |  | |  | |  | |  | |  | |  | |
| **Patient IQR** | | 295-434 | | 219-255 | | 54%-82% | |  | |  | |  | |  | |  | |  | |  | |  | |  | |
| **Normal range** | | 120-246 | | | | | |  | |  | |  | |  | |  | |  | |  | |  | |  | |
| **P value** | | **0.0038** | | | | | |  | |  | |  | |  | |  | |  | |  | |  | |  | |

**P value of a Wilcoxon matched-pairs signed rank test, comparing between the median values of each patient’s Day 1 and Day 28.**

**Table S4. Pro-inflammatory cytokines of COVID-19 patients treated with Allocetra-OTS on Day 1 and Day 28**

|  | **TNF-α (pg/ml)** | | | **IL-1β (pg/ml)** | | | **IL-2 (pg/ml)** | | | **IL-6 (pg/ml)** | | |
| --- | --- | --- | --- | --- | --- | --- | --- | --- | --- | --- | --- | --- |
|  | **Day 1** | **Day 28** | **% change** | **Day 1** | **Day 28** | **% change** | **Day 1** | **Day 28** | **% change** | **Day 1** | **Day 28** | **% change** |
| **Patient 001** | 20.22 | 25.12 | 124% | 1.08 | 3.17 | 294% | 1.86 | 4.09 | 220% | 15.22 | 7.93 | 52% |
| **Patient 002** | 24.34 | 28.25 | 116% | 0.85 | 0.98 | 115% | 10.53 | 15.04 | 143% | 10.47 | 10.61 | 101% |
| **Patient 003** | 30.55 | 25.18 | 82% | 6.7 | 5.19 | 77% | 5.16 | 2.74 | 53% | 27.21 | 2.85 | 10% |
| **Patient 005** | 25.18 | 24.94 | 99% | 2.85 | 1.81 | 64% | 5.08 | 3.04 | 60% | 5.04 | 2.86 | 57% |
| **Patient 006** | 23.73 | 24.76 | 104% | 1.64 | 3.34 | 204% | 10.84 | 10.92 | 101% | 6.51 | 4.47 | 69% |
| **Patient 01-001** | 24.58 | 31.34 | 128% | 0.78 | 0.94 | 121% | 12.75 | 14.12 | 111% | 13.38 | 10.85 | 81% |
| **Patient 01-002** | 27.2 | 23.24 | 85% | 2.14 | 2.72 | 127% | 4.39 | 4.24 | 97% | 14.93 | 6.65 | 45% |
| **Patient 01-003** | 21.88 | 25.66 | 117% | 0.44 | 0.39 | 89% | 2.23 | 1.65 | 74% | 8.98 | 8.18 | 91% |
| **Patient 01-004** | 27.61 | 30.89 | 112% | 1.73 | 1.54 | 89% | 6.12 | 4.62 | 75% | 4.91 | 4.17 | 85% |
| **Patient 01-005** | 93.95 | 30.37 | 32% | 4.81 | 4.33 | 90% | 8.08 | 5.46 | 68% | 10.64 | 7.31 | 69% |
| **Patient 01-007** | 25.42 | 19.83 | 78% | 0.45 | 0.43 | 96% | 1.43 | 0.98 | 69% | 3.06 | 3.42 | 112% |
| **Patient 01-008** | 22.42 | 16.17 | 72% | 1.15 | 0.82 | 71% | 2.66 | 2.08 | 78% | 6.29 | 3.11 | 49% |
| **Patient 01-009** | 26.28 | 16.45 | 63% | 1.13 | 1 | 88% | 2.5 | 2.08 | 83% | 7.92 | 3.42 | 43% |
| **Patient 01-012** | 33.31 | 27.73 | 83% | 1.99 | 2.13 | 107% | 5.33 | 7.67 | 144% | 80.27 | 4.3 | 5% |
| **Patient 01-013** | 20.89 | 20.14 | 96% | 0.84 | 0.77 | 92% | 7.74 | 6.78 | 88% | 3.36 | 4.08 | 121% |
| **Patient 01-014** | 18.17 | 17.28 | 95% | 0.56 | 0.41 | 73% | 2.62 | 1.78 | 68% | 11.8 | 1.02 | 9% |
| **Patient 01-015** | 18.01 | 19.82 | 110% | 1.28 | 1.27 | 99% | 5.02 | 3.7 | 74% | 5.75 | 3.57 | 62% |
| **Patient 02-001** | 21.33 | 13.11 | 61% | 0.5 | 1.41 | 282% | 3.09 | 4.29 | 139% | 2.49 | 2.03 | 82% |
| **Patient 02-002** | 26.28 | 20.84 | 79% | 0.89 | 1.02 | 115% | 3.48 | 3.12 | 90% | 8.2 | 5.89 | 72% |
| **Patient 02-003** | 26.38 | 23.84 | 90% | 1.18 | 1.03 | 87% | 3.45 | 1.11 | 32% | 9.11 | 3.26 | 36% |
| **Patient 03-001** | 32.04 | N/A | N/A | 6.06 | N/A | N/A | 9.72 | N/A | N/A | 12.36 | N/A | N/A |
| **Patient median (N=)** | 25.18 (21) | 24.3 (20) | 93% (20) | 1.15 (21) | 1.15 (20) | 94% (20) | 5.02 (21) | 3.895 (20) | 81% (20) | 8.98 (21) | 4.12 (20) | 65% (20) |
| **Patient IQR** | 21.9-27.2 | 19.8-26.2 | 79%-111% | 0.84-1.99 | 0.91-2.28 | 88%-117% | 2.7-7.7 | 2.1-5.8 | 68%-103% | 5.8-12.4 | 3.2-6.8 | 44%-82% |
| **Control median (N=)** | 14.56 (5) | | | 1.16 (5) | | | 3.27 (5) | | | 3.04 (5) | | |
| **Control IQR** | 13.31-18.13 | | | 0.765-1.32 | | | 3.57-7.62 | | | 2.2-3.97 | | |
| **P value** | **^a^0.0002** | **^a^0.0043** | ^b^0.1054 | ^a^0.6665 | ^a^0.4876 | ^b^0.8908 | ^a^0.3739 | ^a^0.9646 | ^b^0.265 | **^a^0.0018** | ^a^0.1452 | **^b^<0.0001** |
|  | **IL-18 (pg/ml)** | | | **IFN-α (pg/ml)** | | | **IFN-β (pg/ml)** | | | **IFN-γ (pg/ml)** | | |
|  | **Day 1** | **Day 28** | **% change** | **Day 1** | **Day 28** | **% change** | **Day 1** | **Day 28** | **% change** | **Day 1** | **Day 28** | **% change** |
| **Patient 001** | 328.9 | 647.1 | 197% | 0.05 | 4.48 | >1000% | 0.05 | 3.07 | >1000% | 7.84 | 9.37 | 120% |
| **Patient 002** | 1295 | 1016 | 78% | 3.13 | 3.79 | 121% | 2.21 | 2 | 90% | 12.5 | 9.85 | 79% |
| **Patient 003** | 1785 | 1457 | 82% | 10.06 | 4.72 | 47% | 2 | 2.63 | 132% | 16.14 | 8.42 | 52% |
| **Patient 005** | 537.71 | 600.79 | 112% | 4.48 | 5.21 | 116% | 1.43 | 2.63 | 184% | 5.84 | 4.65 | 80% |
| **Patient 006** | 459.26 | 537.71 | 117% | 5.46 | 6.22 | 114% | 2.41 | 2.63 | 109% | 0 | 0 | 100% |
| **Patient 01-001** | 512.2 | 405.01 | 79% | 7.14 | 5.21 | 73% | 3.3 | 2 | 61% | 3.94 | 4.34 | 110% |
| **Patient 01-002** | 253.42 | 140.21 | 55% | 5.46 | 4.25 | 78% | 2 | 2 | 100% | 9.69 | 7.89 | 81% |
| **Patient 01-003** | 474.24 | 266.95 | 56% | 6.74 | 7.54 | 112% | 4.24 | 4.49 | 106% | 6.73 | 3.63 | 54% |
| **Patient 01-004** | 180.15 | 313.31 | 174% | 5.46 | 5.46 | 100% | 2.41 | 2.96 | 123% | 5.99 | 3.34 | 56% |
| **Patient 01-005** | 187.3 | 110.55 | 59% | 3.34 | 5.21 | 156% | 0 | 0 | 100% | 8.84 | 2.75 | 31% |
| **Patient 01-007** | 692.58 | 399.11 | 58% | 0 | 4.02 | >1000% | 7.58 | 18.15 | 239% | 3.2 | 0 | 0% |
| **Patient 01-008** | 364.85 | 248.44 | 68% | 9.97 | 0 | 0% | 0 | 0 | 100% | 11.82 | 0 | 0% |
| **Patient 01-009** | 739.35 | 396.98 | 54% | 83.14 | 3.42 | 4% | 2.73 | 1.99 | 73% | 6.66 | 3.93 | 59% |
| **Patient 01-012** | 777.94 | 1743 | 224% | 8.46 | 3.42 | 40% | 2.35 | 1.99 | 85% | 5.43 | 3.34 | 62% |
| **Patient 01-013** | 788.82 | 381.51 | 48% | 4.96 | 6.08 | 123% | 5.44 | 5.15 | 95% | 2.67 | 3.33 | 125% |
| **Patient 01-014** | 907.82 | 322.27 | 35% | 7.54 | 6.47 | 86% | 4.87 | 4.58 | 94% | 17.12 | 0 | 0% |
| **Patient 01-015** | 455.82 | 362.96 | 80% | 1.68 | 3.59 | 214% | 3.24 | 3.76 | 116% | 10.83 | 8.33 | 77% |
| **Patient 02-001** | 997.69 | 499.71 | 50% | 5.62 | 0 | 0% | 3.54 | 0 | 0% | 0 | 0 | 100% |
| **Patient 02-002** | 329.36 | 439.32 | 133% | 6.73 | 3.59 | 53% | 0 | 0 | 100% | 0 | 0 | 100% |
| **Patient 02-003** | 490.72 | 233.56 | 48% | 6.73 | 5.45 | 81% | 3.76 | 4.31 | 115% | 2.41 | 0 | 0% |
| **Patient 03-001** | 407.19 | N/A | N/A | 5.1 | N/A | N/A | 0 | N/A | N/A | 15.15 | N/A | N/A |
| **Patient median (N=)** | 490.72 (21) | 398 (20) | 73% (20) | 5.46 (21) | 4.6 (20) | 83% (20) | 2.41 (21) | 2.63 (20) | 100% (20) | 6.66 (21) | 3.34 (20) | 69% (20) |
| **Patient IQR** | 365-778 | 302-553 | 55%-113% | 4.5-7.1 | 3.6-5.5 | 49%-116% | 1.4-3.5 | 2.0-3.9 | 92%-115% | 3.2-10.8 | 0.0-5.5 | 47%-100% |
| **Control median (N=)** | 69.3 (5) | | | 0 (5) | | | 0 (9) | | | 0 (6) | | |
| **Control IQR** | 47-109 | | | 0-0 | | | 0-0 | | | 0-0 | | |
| **P value** | **^a^<0.0001** | **^a^<0.0001** | ^b^0.0532 | **^a^0.0002** | ^a^0.0008 | ^b^0.1956 | **^a^0.0001** | **^a^0.0002** | ^b^0.5198 | **^a^0.0006** | **^a^0.0135** | **^b^0.0003** |

1. **P value of a non-parametric two-tailed Mann-Whitney test, comparing between median values of COVID-19 patients and healthy individuals.**
2. **P value of a Wilcoxon matched-pairs signed rank test, comparing between the median values of each patient’s Day 1 and Day 28.**

**Table S5. Anti-inflammatory cytokines of COVID-19 patients treated with Allocetra-OTS on Day 1 and Day 28**

|  | **IL-10 (pg/ml)** | | | **IL-1Ra (pg/ml)** | | | **IL-2Rα (pg/ml)** | | | **TNFRI (pg/ml)** | | |
| --- | --- | --- | --- | --- | --- | --- | --- | --- | --- | --- | --- | --- |
|  | **Day 1** | **Day 28** | **% change** | **Day 1** | **Day 28** | **% change** | **Day 1** | **Day 28** | **% change** | **Day 1** | **Day 28** | **% change** |
| **Patient 001** | 4.94 | 1.82 | 37% | 1458 | 1194 | 82% | 672.15 | 719.1 | 107% | 683.63 | 1248 | 183% |
| **Patient 002** | 8.3 | 1.07 | 13% | 1268 | 651.38 | 51% | 2216 | 1531 | 69% | 2445 | 1937 | 79% |
| **Patient 003** | 18.28 | 1.3 | 7% | 3903 | 756.17 | 19% | 1402 | 805.17 | 57% | 2696 | 1354 | 50% |
| **Patient 005** | 3.03 | 2.75 | 91% | 1978 | 1309 | 66% | 1153 | 996.55 | 86% | 2686 | 1908 | 71% |
| **Patient 006** | 2.34 | 1.23 | 53% | 867.13 | 843.02 | 97% | 1050 | 741.29 | 71% | 2482 | 1473 | 59% |
| **Patient 01-001** | 4.65 | 3.18 | 68% | 1455 | 1494 | 103% | 1179 | 1072 | 91% | 3253 | 2296 | 71% |
| **Patient 01-002** | 5.52 | 0.98 | 18% | 3029 | 2385 | 79% | 532.47 | 301.86 | 57% | 2037 | 1077 | 53% |
| **Patient 01-003** | 2.54 | 1.24 | 49% | 794.68 | 1464 | 184% | 888.79 | 661.7 | 74% | 2639 | 2359 | 89% |
| **Patient 01-004** | 3.56 | 1.88 | 53% | 1693 | 1219 | 72% | 607.25 | 623.12 | 103% | 2278 | 2144 | 94% |
| **Patient 01-005** | 4.02 | 2.1 | 52% | 993.54 | 387.1 | 39% | 385.07 | 333.84 | 87% | 3016 | 1997 | 66% |
| **Patient 01-007** | 4.13 | 2.83 | 69% | 1039 | 832.03 | 80% | 1509 | 1002 | 66% | 2163 | 2157 | 100% |
| **Patient 01-008** | 31.18 | 0.49 | 2% | 1918 | 855.16 | 45% | 1133 | 615.17 | 54% | 2268 | 1237 | 55% |
| **Patient 01-009** | 20.22 | 2.26 | 11% | 2391 | 1285 | 54% | 1096 | 774.33 | 71% | 2509 | 1682 | 67% |
| **Patient 01-012** | 8.31 | 6.99 | 84% | 4942 | 2652 | 54% | 2657 | 2654 | 100% | 5218 | 10924 | 209% |
| **Patient 01-013** | 4.52 | 1.64 | 36% | 1473 | 1221 | 83% | 720.25 | 470.88 | 65% | 1546 | 1365 | 88% |
| **Patient 01-014** | 9.83 | 0.4 | 4% | 1438 | 580.85 | 40% | 1121 | 619.73 | 55% | 2348 | 1672 | 71% |
| **Patient 01-015** | 5.79 | 0.21 | 4% | 622.74 | 1101 | 177% | 801.26 | 532.18 | 66% | 1409 | 1114 | 79% |
| **Patient 02-001** | 4.15 | 2.85 | 69% | 496.09 | 1493 | 301% | 1102 | 551.9 | 50% | 1897 | 1413 | 74% |
| **Patient 02-002** | 5.77 | 4.27 | 74% | 851.23 | 888.43 | 104% | 825.29 | 1256 | 152% | 3785 | 4247 | 112% |
| **Patient 02-003** | 6.66 | 0.43 | 6% | 774.71 | 949.62 | 123% | 1247 | 936.87 | 75% | 3277 | 2433 | 74% |
| **Patient 03-001** | 8.65 | N/A | N/A | 743.65 | N/A | N/A | 714.39 | N/A | N/A | 2525 | N/A | N/A |
| **Patient median (N=)** | 5.52 (21) | 1.73 (20) | 43% (20) | 1438 (21) | 1147 (20) | 79% (20) | 1096 (21) | 730 (20) | 71% (20) | 2482 (21) | 1795 (20) | 74% (20) |
| **Patient IQR** | 4.1-8.3 | 1.0-2.8 | 10%-68% | 851-1,918 | 840-1,348 | 53%-103% | 720-1,179 | 599-998 | 63%-88% | 2,163-2,696 | 1,362-2,192 | 67%-91% |
| **Control median (N=)** | 0.89 (5) | | | 342 (5) | | | 183 (5) | | | 420.7 (5) | | |
| **Control IQR** | 0.8-1.1 | | | 302-369 | | | 156-197 | | | 364-811 | | |
| **P value** | **^a^<0.0001** | ^a^0.0832 | **^b^<0.0001** | **^a^<0.0001** | **^a^<0.0001** | **^b^0.0328** | **^a^<0.0001** | **^a^<0.0001** | **^b^0.0006** | **^a^0.0001** | **^a^<0.0001** | **^b^0.0073** |
|  | **IL-4 (pg/ml)** | | |  | | |  | | |  | | |
|  | **Day 1** | **Day 28** | **% change** |  |  |  |  |  |  |  |  |  |
| **Patient 001** | 4.78 | 6.54 | 137% |  |  |  |  |  |  |  |  |  |
| **Patient 002** | 3 | 1.54 | 51% |  |  |  |  |  |  |  |  |  |
| **Patient 003** | 1.54 | 1.42 | 92% |  |  |  |  |  |  |  |  |  |
| **Patient 005** | 4.24 | 4.08 | 96% |  |  |  |  |  |  |  |  |  |
| **Patient 006** | 11.99 | 3.7 | 31% |  |  |  |  |  |  |  |  |  |
| **Patient 01-001** | 2.18 | 0.26 | 12% |  |  |  |  |  |  |  |  |  |
| **Patient 01-002** | 1.97 | 0.31 | 16% |  |  |  |  |  |  |  |  |  |
| **Patient 01-003** | 2.65 | 2.67 | 101% |  |  |  |  |  |  |  |  |  |
| **Patient 01-004** | 0.37 | 0 | 0% |  |  |  |  |  |  |  |  |  |
| **Patient 01-005** | 0.53 | 0 | 0% |  |  |  |  |  |  |  |  |  |
| **Patient 01-007** | 1.59 | 0.22 | 14% |  |  |  |  |  |  |  |  |  |
| **Patient 01-008** | 5.51 | 3.33 | 60% |  |  |  |  |  |  |  |  |  |
| **Patient 01-009** | 0.15 | 1.06 | 707% |  |  |  |  |  |  |  |  |  |
| **Patient 01-012** | 0.42 | 0 | 0% |  |  |  |  |  |  |  |  |  |
| **Patient 01-013** | 4.91 | 1.43 | 29% |  |  |  |  |  |  |  |  |  |
| **Patient 01-014** | 21.37 | 6.68 | 31% |  |  |  |  |  |  |  |  |  |
| **Patient 01-015** | 37.9 | 40.56 | 107% |  |  |  |  |  |  |  |  |  |
| **Patient 02-001** | 16.61 | 4.73 | 28% |  |  |  |  |  |  |  |  |  |
| **Patient 02-002** | 1.76 | 1.03 | 59% |  |  |  |  |  |  |  |  |  |
| **Patient 02-003** | 1.09 | 0 | 0% |  |  |  |  |  |  |  |  |  |
| **Patient 03-001** | 2.29 | N/A | N/A |  |  |  |  |  |  |  |  |  |
| **Patient median (N=)** | 2.29 (21) | 1.43 (20) | 31% (20) |  |  |  |  |  |  |  |  |  |
| **Patient IQR** | 1.5-4.9 | 0.3-3.8 | 13%-93% |  |  |  |  |  |  |  |  |  |
| **Control median (N=)** | 0.0 (5) | | |  | | |  | | |  | | |
| **Control IQR** | 0-0.175 | | |  | | |  | | |  | | |
| **P value** | **^a^<0.0001** | **^a^0.0139** | **^b^0.0107** |  |  |  |  |  |  |  |  |  |

1. **P value of a non-parametric two-tailed Mann-Whitney test, comparing between median values of COVID-19 patients and healthy individuals.**
2. **P value of a Wilcoxon matched-pairs signed rank test, comparing between the median values of each patient’s Day 1 and Day 28.**

**Table S6. Chemokines of COVID-19 patients treated with Allocetra-OTS on Day 1 and Day 28**

|  | **MCP-1 (pg/ml)** | | | **MIP-1α (pg/ml)** | | | **IP-10 (pg/ml)** | | | **IL-8 (pg/ml)** | | |
| --- | --- | --- | --- | --- | --- | --- | --- | --- | --- | --- | --- | --- |
|  | **Day 1** | **Day 28** | **% change** | **Day 1** | **Day 28** | **% change** | **Day 1** | **Day 28** | **% change** | **Day 1** | **Day 28** | **% change** |
| **Patient 001** | 497.27 | 592.48 | 119% | 0 | 4.83 | >200% | 33.58 | 86.79 | 258% | 19.26 | 19.67 | 102% |
| **Patient 002** | 293.21 | 378.19 | 129% | 6.75 | 9.51 | 141% | 734.8 | 73.64 | 10% | 29.29 | 16.27 | 56% |
| **Patient 003** | 796.15 | 464.19 | 58% | 22.86 | 16.6 | 73% | 85.55 | 78.87 | 92% | 53.99 | 28.89 | 54% |
| **Patient 005** | 299.25 | 364.17 | 122% | 17.57 | 27.06 | 154% | 73.27 | 63.54 | 87% | 24.41 | 31.64 | 130% |
| **Patient 006** | 280.62 | 371.34 | 132% | 8.83 | 11.27 | 128% | 22.69 | 48.71 | 215% | 14.89 | 21.61 | 145% |
| **Patient 01-001** | 290.38 | 563.71 | 194% | 0 | 10.83 | >1000% | N/A | N/A | N/A | 18.14 | 30.29 | 167% |
| **Patient 01-002** | 258.82 | 252.91 | 98% | 0 | 0 | 100% | N/A | N/A | N/A | 21.18 | 18.47 | 87% |
| **Patient 01-003** | 259.1 | 668.13 | 258% | 0 | 8.61 | >1000% | N/A | N/A | N/A | 34.25 | 46.34 | 135% |
| **Patient 01-004** | 191.22 | 376.38 | 197% | 10.4 | 13.38 | 129% | N/A | N/A | N/A | 38.57 | 45.19 | 117% |
| **Patient 01-005** | 469.1 | 317.74 | 68% | 0 | 0 | 100% | N/A | N/A | N/A | 19.44 | 15.39 | 79% |
| **Patient 01-007** | 229.81 | 368.58 | 160% | 0 | 12.42 | >1000% | 189.03 | 80.21 | 42% | 22.88 | 14.5 | 63% |
| **Patient 01-008** | 262.25 | 324.62 | 124% | 0 | 0 | 100% | 463.96 | 22.18 | 5% | 37.21 | 17.15 | 46% |
| **Patient 01-009** | 412.85 | 326.72 | 79% | 14.58 | 10.16 | 70% | 710.95 | 103.65 | 15% | 32.07 | 15.99 | 50% |
| **Patient 01-012** | 679.57 | 363.32 | 53% | 10.93 | 0 | 0% | 2755 | 590.11 | 21% | 43.45 | 40.28 | 93% |
| **Patient 01-013** | 210.99 | 428.26 | 203% | 0 | 0 | 100% | 628.37 | 143.59 | 23% | 15.08 | 14.29 | 95% |
| **Patient 01-014** | 490.86 | 623.37 | 127% | 7.96 | 0 | 0% | 1081 | 82.55 | 8% | 52.18 | 26.27 | 50% |
| **Patient 01-015** | 382.82 | 841.04 | 220% | 0 | 7.72 | >1000% | 144.84 | 85.03 | 59% | 17.7 | 26.52 | 150% |
| **Patient 02-001** | 324.02 | 299.61 | 92% | 0 | 0 | 100% | 675.26 | 12.18 | 2% | 28.17 | 20.9 | 74% |
| **Patient 02-002** | 377.59 | 422.36 | 112% | 0 | 0 | 100% | 585.12 | 67.55 | 12% | 28.23 | 23.18 | 82% |
| **Patient 02-003** | 1081 | 489.03 | 45% | 0 | 0 | 100% | 1285 | 82.63 | 6% | 50.48 | 29.62 | 59% |
| **Patient 03-001** | 626.62 | N/A | N/A | 0 | N/A | N/A | 405.97 | N/A | N/A | 58.07 | N/A | N/A |
| **Patient median (N=)** | 324 (21) | 377 (20) | 123% (20) | 0 (21) | 6.27 (20) | 100% (20) | 524 (16) | 80.21 (15) | 21% (15) | 28.23 (21) | 22.39 (20) | 85% (20) |
| **Patient IQR** | 262-491 | 354-508 | 89%-169% | 0.00-8.83 | 0.00-10.94 | 86%-114% | 130-717 | 66-86 | 9%-73% | 19.4-38.6 | 16.9-29.8 | 58%-120% |
| **Control median (N=)** | 249 (5) | | | 0 (10) | | | 24.8 (5) | | | 9.39 (5) | | |
| **Control IQR** | 200.7-272.5 | | | 0-0 | | | 23.4-27.7 | | | 7.44-14.62 | | |
| **P value** | ^a^**0.0234** | ^a^**0.0002** | ^b^0.2774 | **^a^0.0438** | **^a^0.0062** | ^b^0.3054 | **^a^0.0019** | **^a^0.0146** | **^b^0.0012** | ^a^**<0.0001** | ^a^**0.0002** | ^b^0.1327 |
|  | **MCP-3 (pg/ml)** | | | **MIG (pg/ml)** | | |  |  |  |  |  |  |
|  | **Day 1** | **Day 28** | **% change** | **Day 1** | **Day 28** | **% change** |  |  |  |  |  |  |
| **Patient 001** | 135.17 | 107.45 | 79% | 6562 | 1365 | 21% |  |  |  |  |  |  |
| **Patient 002** | 16.94 | 5.57 | 33% | 1691 | 1749 | 103% |  |  |  |  |  |  |
| **Patient 003** | 12.7 | 9.17 | 72% | 3440 | 2598 | 76% |  |  |  |  |  |  |
| **Patient 005** | 13.99 | 10.51 | 75% | 1670 | 2394 | 143% |  |  |  |  |  |  |
| **Patient 006** | 0 | 0 | 100% | 1140 | 1659 | 146% |  |  |  |  |  |  |
| **Patient 01-001** | 0 | 11.3 | >1000% | 3824 | 6570 | 172% |  |  |  |  |  |  |
| **Patient 01-002** | 17.38 | 0 | 0% | 2752 | 3407 | 124% |  |  |  |  |  |  |
| **Patient 01-003** | 0 | 0 | 100% | 5921 | 3614 | 61% |  |  |  |  |  |  |
| **Patient 01-004** | 18.46 | 7.32 | 40% | 13637 | 3867 | 28% |  |  |  |  |  |  |
| **Patient 01-005** | 80.26 | 44.14 | 55% | 1720 | 1108 | 64% |  |  |  |  |  |  |
| **Patient 01-007** | 0 | 0 | 100% | 6415 | 4678 | 73% |  |  |  |  |  |  |
| **Patient 01-008** | 13 | 0 | 0% | 7471 | 2758 | 37% |  |  |  |  |  |  |
| **Patient 01-009** | 23.8 | 5.59 | 23% | 7777 | 5694 | 73% |  |  |  |  |  |  |
| **Patient 01-012** | 26.49 | 10.31 | 39% | 16872 | 19908 | 118% |  |  |  |  |  |  |
| **Patient 01-013** | 184.28 | 112.17 | 61% | 7569 | 6100 | 81% |  |  |  |  |  |  |
| **Patient 01-014** | 76.25 | 0 | 0% | 27807 | 6013 | 22% |  |  |  |  |  |  |
| **Patient 01-015** | 114.29 | 139.05 | 122% | 6324 | 6998 | 111% |  |  |  |  |  |  |
| **Patient 02-001** | 46.4 | 13.38 | 29% | 4064 | 2126 | 52% |  |  |  |  |  |  |
| **Patient 02-002** | 0 | 0 | 100% | 7856 | 9751 | 124% |  |  |  |  |  |  |
| **Patient 02-003** | 51.96 | 18 | 35% | 21489 | 13242 | 62% |  |  |  |  |  |  |
| **Patient 03-001** | 26.79 | N/A | N/A | 13075 | N/A |  |  |  |  |  |  |  |
| **Patient median (N=)** | 18.46 (21) | 8.24 (20) | 55% (20) | 6415 (21) | 3740 (20) | 74% (20) |  |  |  |  |  |  |
| **Patient IQR** | 12.7-52.0 | 0.0-14.5 | 31%-90% | 3,440-7,856 | 2,327-6,218 | 59%-119% |  |  |  |  |  |  |
| **Control median (N=)** | 0 (6) | | | 1171 (5) | | |  |  |  |  |  |  |
| **Control IQR** | 0-0 | | | 839-1251 | | |  |  |  |  |  |  |
| **P value** | ^a^**0.0032** | ^a^**0.0138** | **^b^0.0034** | **^a^0.0002** | **^a^0.0003** | ^b^0.0759 |  |  |  |  |  |  |

1. **P value of a non-parametric two-tailed Mann-Whitney test, comparing between median values of COVID-19 patients and healthy individuals.**
2. **P value of a Wilcoxon matched-pairs signed rank test, comparing between the median values of each patient’s Day 1 and Day 28.**

**Table S7. Hematopoietic growth factors of COVID-19 patients treated with Allocetra-OTS on Day 1 and Day 28**

|  | **G-CSF (pg/ml)** | | | **IL-7 (pg/ml)** | | | **GM-CSF (pg/ml)** | | |
| --- | --- | --- | --- | --- | --- | --- | --- | --- | --- |
|  | **Day 1** | **Day 28** | **% change** | **Day 1** | **Day 28** | **% change** | **Day 1** | **Day 28** | **% change** |
| **Patient 001** | 9.29 | 18.75 | 202% | 5.01 | 5.24 | 105% | 96.68 | 106.48 | 110% |
| **Patient 002** | 15.55 | 7.68 | 49% | 29.24 | 8.72 | 30% | 0 | 0 | 100% |
| **Patient 003** | 58.6 | 18.1 | 31% | 9.15 | 7.05 | 77% | 0 | 0 | 100% |
| **Patient 005** | 11.21 | 11.82 | 105% | 11.96 | 8.94 | 75% | 8.97 | 3.82 | 43% |
| **Patient 006** | 9.71 | 12.13 | 125% | 26.22 | 14.38 | 55% | 0 | 0 | 100% |
| **Patient 01-001** | 20.04 | 15.55 | 78% | 17.3 | 8.3 | 48% | 0 | 0 | 100% |
| **Patient 01-002** | 23.29 | 15.55 | 67% | 12.61 | 8.09 | 64% | 0 | 0 | 100% |
| **Patient 01-003** | 11.82 | 11.82 | 100% | 19.11 | 8.3 | 43% | 0 | 0 | 100% |
| **Patient 01-004** | 16.19 | 20.69 | 128% | 14.83 | 5.64 | 38% | 0 | 0 | 100% |
| **Patient 01-005** | 17.46 | 10.61 | 61% | 13.72 | 8.3 | 60% | 0 | 13.05 | >1000% |
| **Patient 01-007** | 0 | 13.04 | >1000% | 25.83 | 10 | 39% | 0 | 0 | 100% |
| **Patient 01-008** | 26.4 | 13.04 | 49% | 17.42 | 10.53 | 60% | 0 | 0 | 100% |
| **Patient 01-009** | 31.24 | 11.2 | 36% | 14.82 | 6.9 | 47% | 0 | 0 | 100% |
| **Patient 01-012** | 102 | 10.75 | 11% | 15.1 | 4.23 | 28% | 0 | 0 | 100% |
| **Patient 01-013** | 18.73 | 17.01 | 91% | 16.6 | 6.1 | 37% | 6.87 | 10.17 | 148% |
| **Patient 01-014** | 22.93 | 10.07 | 44% | 30.21 | 10.02 | 33% | 0 | 0 | 100% |
| **Patient 01-015** | 23.64 | 12.64 | 53% | 15.36 | 9.78 | 64% | 112.19 | 549.1 | 489% |
| **Patient 02-001** | 16.79 | 5.15 | 31% | 31.52 | 5.91 | 19% | 0 | 0 | 100% |
| **Patient 02-002** | 22.23 | 16.67 | 75% | 12.67 | 5.66 | 45% | 0 | 0 | 100% |
| **Patient 02-003** | 10.7 | 20.82 | 195% | 18.84 | 10.5 | 56% | 0 | 0 | 100% |
| **Patient 03-001** | 5.42 | N/A | N/A | 23.24 | N/A | N/A | 0 | N/A | N/A |
| **Patient median (N=)** | 17.46 (21) | 12.84 (20) | 67% (19) | 16.6 (21) | 8.3 (20) | 47% (19) | 0 (19) | 0 (18) | 100% (17) |
| **Patient IQR** | 11.2-23.3 | 11.1-16.8 | 47%-103% | 13.7-23.2 | 6.1-9.8 | 38%-61% | 0.00-0.00 | 0.00-0.96 | 100%-100% |
| **Control median (N=)** | 0 (5) | | | 4.32 (6) | | | 0 (9) | | |
| **Control IQR** | 0-2.235 | | | 3.3-4.55 | | | 0-0 | | |
| **P value** | **^a^0.0002** | **^a^<0.0001** | **^b^0.0446** | **^a^<0.0001** | **^a^<0.0001** | **^b^<0.0001** | ^a^0.2874 | ^a^0.1797 | ^b^0.75 |

1. **P value of a non-parametric two-tailed Mann-Whitney test, comparing between median values of COVID-19 patients and healthy individuals.**
2. **P value of a Wilcoxon matched-pairs signed rank test, comparing between the median values of each patient’s Day 1 and Day 28.**

**
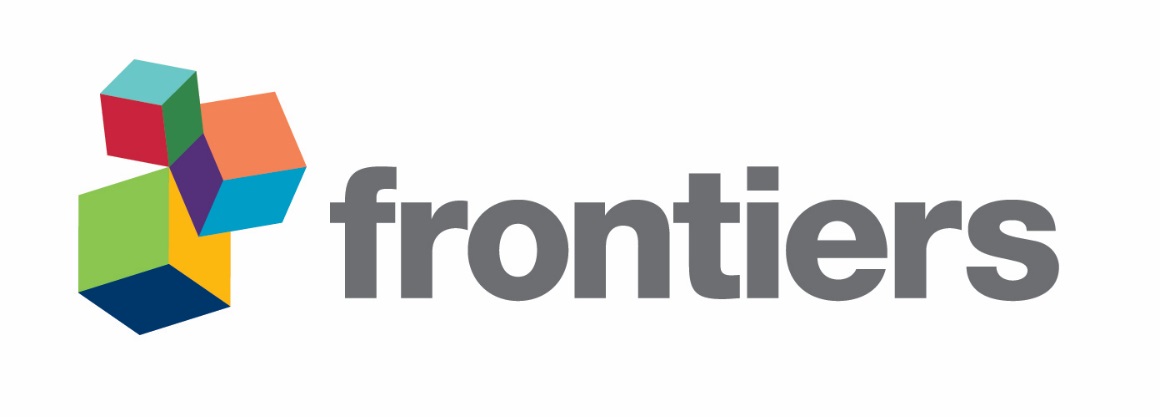
**
